# Capture, Release, and Identification of Newly Synthesized Proteins for Improved Profiling of Functional Translatomes

**DOI:** 10.1016/j.mcpro.2023.100497

**Published:** 2023-01-13

**Authors:** Nancy J. Phillips, Bala M. Vinaithirthan, Juan A. Oses-Prieto, Robert J. Chalkley, Alma L. Burlingame

**Affiliations:** 1 Department of Pharmaceutical Chemistry, University of California, San Francisco, San Francisco, California, USA; 2 Helen Diller Family Comprehensive Cancer Center, University of California, San Francisco, San Francisco, California, USA

**Keywords:** nascent proteins, translation, O-propargyl-puromycin, cleavable linker, mTOR, AHA, L-azidohomoalanine, BCA, bicinchoninic acid, cRAP, common Repository of Adventitious Proteins, CRAPome, Contaminant Repository for Affinity Purification, DMSO, dimethyl sulfoxide, FC, fold change, GO, Gene Ontology, HPG, L-homopropargylglycine, LC-MS/MS, liquid chromatography–tandem mass spectrometry, mTOR, mammalian target of rapamycin, MS, mass spectrometry, OPP, O-propargyl-puromycin, OPP-ID, OPP-mediated identification, OPP-ID_CL_, OPP-ID using a cleavable biotin-azide linker, RBP, RNA-binding protein, SILAC, stable isotope labeling by amino acids in cell culture

## Abstract

New protein synthesis is regulated both at the level of mRNA transcription and translation. RNA-Seq is effective at measuring levels of mRNA expression, but techniques to monitor mRNA translation are much more limited. Previously, we reported results from O-propargyl-puromycin (OPP) labeling of proteins undergoing active translation in a 2-h time frame, followed by biotinylation using click chemistry, affinity purification, and on-bead digestion to identify nascent proteins by mass spectrometry (OPP-ID). As with any on-bead digestion protocol, the problem of nonspecific binders complicated the rigorous categorization of nascent proteins by OPP-ID. Here, we incorporate a chemically cleavable linker, Dde biotin-azide, into the protocol (OPP-ID_CL_) to provide specific release of modified proteins from the streptavidin beads. Following capture, the Dde moiety is readily cleaved with 2% hydrazine, releasing nascent polypeptides bearing OPP plus a residual C_3_H_8_N_4_ tag. When results are compared side by side with the original OPP-ID method, change to a cleavable linker led to a dramatic reduction in the number of background proteins detected in controls and a concomitant increase in the number of proteins that could be characterized as newly synthesized. We evaluated the method’s ability to detect nascent proteins at various submilligram protein input levels and showed that, when starting with only 100 μg of protein, ∼1500 nascent proteins could be identified with low background. Upon treatment of K562 cells with MLN128, a potent inhibitor of the mammalian target of rapamycin, prior to OPP treatment, we identified 1915 nascent proteins, the majority of which were downregulated upon inhibitor treatment. Repressed proteins with log2 FC <-1 revealed a complex network of functionally interacting proteins, with the largest cluster associated with translational initiation. Overall, incorporation of the Dde biotin-azide cleavable linker into our protocol has increased the depth and accuracy of profiling of nascent protein networks.

## Highlights

• O-propargyl-puromycin was used to capture and identify proteins during active translation.
• Cleavable linker Dde biotin-azide permitted selective release of nascent proteins.
• Dramatic increase in the observed global nascent proteome was achieved.
• Upon acute inhibition of mTOR, a network of downregulated proteins was identified.

## Graphical Abstract

**Figure:**
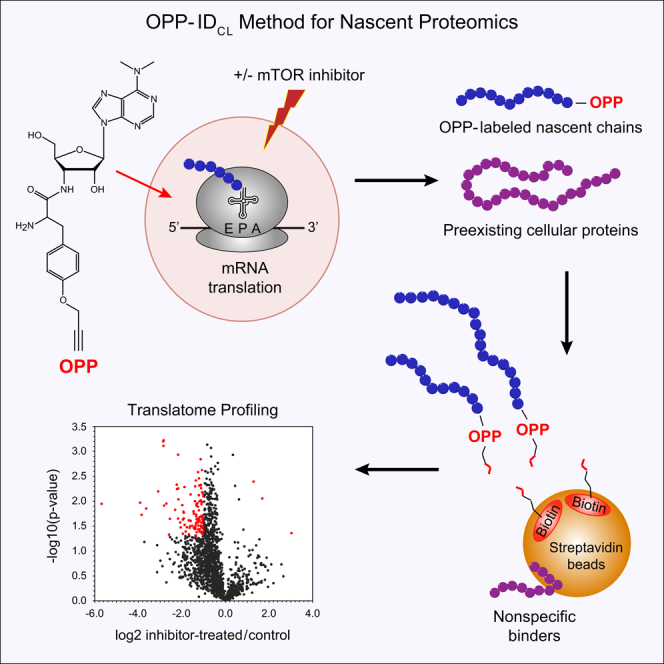

## In Brief

In this study, we have optimized the O-propargyl-puromycin (OPP)-mediated identification of nascent proteins by incorporating a cleavable linker, Dde biotin-azide, into our protocol. Following capture on streptavidin agarose resin, nascent polypeptides were selectively released from the resin by treatment with 2% hydrazine. Using the cleavable linker reduced the presence of background proteins and facilitated the detection of low-abundance proteins. We profiled the nascent proteome upon acute inhibition of mTOR and observed downregulation of many proteins involved in translational initiation.

In the flow of genetic information from DNA to mRNA to proteins, the regulation of protein translation is an essential factor determining the composition of the cellular proteome. Upon stress or exposure to external stimuli, dynamic alterations in the translatome can be triggered, impacting cell fate. Such newly translated proteins are markers of cellular responses and provide insights into the biochemical processes involved. Our ability to characterize nascent proteins accurately and quantitatively is key to our understanding of regulation and dysregulation of protein synthesis in cell development and disease.

A variety of analytical methods exist to study nascent proteomes (1, 2, 3). Pulsed stable isotope labeling by amino acids in cell culture (SILAC) or biosynthetic incorporation of bioorthogonal, noncanonical amino acids, such as L-azidohomoalanine (AHA) and L-homopropargylglycine (HPG), capture translation during a defined time frame (4, 5, 6, 7). In combination, these labeling methods have been used for quantitative analysis of nascent proteomes (QuaNCAT) (8). Another recent strategy involves heavy isotope labeling of AHA for quantitative analysis (9). One drawback of such experiments is that they need to be conducted in media lacking the natural amino acid to be replaced, which is not always practical in complex model systems. The recent pairing of pulsed SILAC with isobaric tandem mass tag labeling in the presence of a booster channel, termed multiplexed enhanced protein dynamics (mePROD) proteomics, represents another option for quantitative analysis of nascent proteomes where multiplexing is possible (10).

As an alternative to metabolic labeling with amino acid analogues, nascent polypeptide chains undergoing elongation can be covalently tagged with the protein synthesis inhibitor puromycin, an aminonucleoside antibiotic whose chemical structure resembles the 3′ end of a tyrosine tRNA (11, 12). Puromycin can enter the A-site in the ribosome and get incorporated randomly at the C terminus of polypeptide chains, leading to premature chain termination and release of truncated, puromycylated polypeptides. Nascent chains can also be labeled in purified, intact ribosome-nascent chain complexes using a biotinylated form of puromycin (PUNCH-P) (13). A recent review article summarizes the current scope of puromycin research and insights generated from laboratory and clinical studies (14).

The puromycin analogue O-propargyl-puromycin (OPP), first introduced in 2012 (15), not only tags nascent proteins but also bears a bioorthogonal alkyne moiety useful for subsequent click chemistry with azides. Using OPP, we previously developed a mass spectrometry (MS)-based proteomic strategy to identify and quantify nascent proteins involving OPP labeling, click chemistry with biotin-azide, affinity purification, and on-bead digestion, which we termed OPP-ID (16). Others have further modified OPP to generate “caged” forms that are incapable of incorporation into elongating polypeptide chains until uncaged in the presence of uncaging enzymes expressed in the target cells of interest (17).

Regardless of which labeling approach is utilized to tag nascent proteins with a clickable handle (OPP, AHA, or HPG), after the appropriate click reaction, affinity purification on functionalized agarose resin or magnetic beads is typically required. Even with stringent washing conditions, the problem of nonspecific binders (“sticky proteins”) appearing in negative controls cannot be fully overcome by washing steps alone. To address this issue, the online Contaminant Repository for Affinity Purification (CRAPome) was created to catalog nonspecific binders frequently observed in affinity purifications (18). This resource is a valuable research tool but does not mitigate the problem.

OPP labeling was recently combined with pulsed SILAC labeling as a strategy to overcome the background problem in affinity purifications, while also facilitating quantification of nascent proteins by SILAC (19). To avoid the use of click chemistry, a modification of this method was introduced that involves labeling with puromycin itself and enrichment of nascent polypeptide chains by immunoprecipitation with an anti-puromycin antibody (20). While promising as solutions to the background problem, these methods do require the dual-pulse labeling with SILAC in conjunction with the OPP or puromycin labeling, in addition to any other experimental treatments being conducted. As an alternative to metabolic labeling, a workflow for quantitative OPP tagging that incorporates tandem mass tag labeling has also been described to address the limitations of methodologies requiring SILAC (21).

**Fig. 1:**
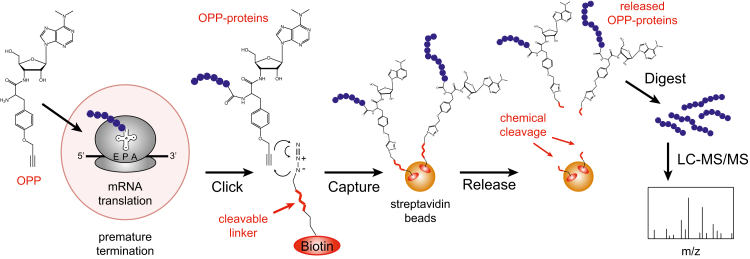
**Schematic diagram showing incorporation of a cleavable biotin-azide linker into our OPP-ID protocol for OPP-tagging, capture, and detection of nascent proteins.** Upon chemical cleavage of the linker, nascent proteins are selectively released, subjected to in-solution digestion, and analyzed by liquid chromatography–tandem mass spectrometry (LC-MS/MS). OPP, O-propargyl-puromycin.

The goal of our present study was to optimize detection and identification of nascent proteins in OPP capture experiments from minimal amounts of starting protein. To this end, we investigated the use of cleavable biotin-azide linkers in the OPP-ID protocol (Fig. 1). Theoretically, this should reduce the problem of nonspecific binders and permit in-solution digestion of clean, released nascent protein pools. In other types of MS-based experiments involving affinity purifications, introduction of a cleavable linker has proven valuable in lowering background and facilitating the identification of targets with higher confidence (22). Indeed, we found a significant reduction in the levels of background proteins using cleavable Dde biotin-azide as compared with regular biotin-azide with on-bead digestion, even at low protein input levels. By diminishing the ambiguity in nascent protein identifications, we were able to comprehensively characterize changes to the translatome upon acute inhibition of the mammalian target of rapamycin (mTOR) with an ATP-competitive inhibitor targeting mTOR in both the mTORC1 and mTORC2 protein complexes.

## Materials and Methods

### Cell Culture

K562 cells were cultured in RPMI media 1640-GlutaMax (Gibco, Thermo Fisher Scientific) supplemented with 10% fetal bovine serum (Life Technologies, Thermo Fisher Scientific), 100 U/ml penicillin, and 100 μg/ml streptomycin (Pen Strep; Gibco, Thermo Fisher Scientific) in T75 flasks. Cells were maintained in a humidified 37 °C incubator with 5% CO_2_. Culture density was kept at <2 × 10^6^/ml. The K562 cell line originated from a patient with chronic myelogenous leukemia.

### Treatment of Cells With Inhibitor and/or OPP

For labeling experiments, K562 cells were cultured to a density of ∼1 × 10^6^/ml, typically in a total of 28 ml of media. For simple labeling of nascent proteomes with OPP, cells were treated with either PBS vehicle or 30 μM OPP (Jena Biosciences) for 2 h in the full medium at 37 °C in the incubator, with periodic gentle agitation of the flasks. For analysis of mTOR-dependent nascent proteomes, K562 cells were treated with either dimethyl sulfoxide (DMSO) vehicle (0.1%, v/v) or 300 nM MLN128 (Cell Signaling Technology) for 1 h prior to the 2-h OPP treatment. After treatment, cells were collected by centrifugation at 300*g* for 5 min at 4 °C, washed 2 times with 10 ml of ice-cold PBS, moved to 1.5-ml Eppendorf tubes, and washed two more times with 1 ml of ice-cold PBS. Cell pellets were frozen at −80 °C until ready for lysis.

### Preparation of Cell Lysates

Cell pellets were resuspended in lysis buffer containing 100 mM Hepes pH 7.5, 150 mM NaCl, 1% Nonidet P-40, 2 mM PMSF, and 1× complete EDTA-free protease inhibitor cocktail (Roche, Millipore Sigma). Lysates were mixed thoroughly by pipetting up and down with 1-mL Eppendorf pipette tips and then allowed to sit on ice for 30 min. Cellular debris was removed by centrifugation at 14,000*g* for 30 min at 4 °C. Protein concentrations were determined by bicinchoninic acid (BCA) assay (Bio-Rad), and cell lysates were diluted with lysis buffer to normalize final protein concentrations, typically to ∼3 mg/ml. Portions of the samples to be reacted with biotin-azide for affinity purification using the original OPP-ID protocol (16) were precleared by overnight incubation with streptavidin agarose resin (Thermo Scientific) at 4 °C with slow rotation prior to click chemistry.

### Preparation of Whole Cellular Proteomes

Portions of the lysates from cells treated with either DMSO or MLN128 prior to OPP treatment (400 μg protein each) were treated with five volumes of ice-cold acetone and allowed to sit at −20 °C overnight. Precipitated proteins were pelleted by centrifugation at 20,000*g* at 4 °C for 10 min and washed twice with ice-cold acetone, with 30-min precipitations at −20 °C and centrifugation as above. The final pellets were briefly air dried and then resolubilized in 40 μl of 8 M guanidine HCl. Samples were then diluted to 6 M guanidine HCl with 80 mM ammonium bicarbonate for reduction with 5 mM DTT at 56 °C for 30 min, alkylation with 20 mM iodoacetamide at room temperature (RT) for 15 min in the dark, and final treatment with 5 mM DTT for 5 min. Following dilution to <1 M guanidine HCl with 80 mM ammonium bicarbonate, samples were incubated at 37 °C on a shaking incubator with 3 μg of Lys C (Fujifilm Wako Pure Chemical Corporation) for 6 h, followed by two overnight digestions with 4 and 8 μg of MS-grade trypsin (Thermo Scientific). Digests were then quenched with 1% formic acid and aliquots enriched on C18 Omix Tips (Agilent Technologies) and eluted with 50% acetonitrile containing 0.1% formic acid. The samples were dried by SpeedVac and then resuspended in 0.1% formic acid for analysis by liquid chromatography–tandem mass spectrometry (LC-MS/MS).

### Click Conjugation of OPP-Labeled Proteins with TAMRA-Azide, Biotin-Azide, Diazo Biotin-Azide, or Dde Biotin-Azide

For click chemistry, a reagent mix was freshly prepared by sequential addition of stock solutions to obtain 6.25% SDS, 0.625 mM azide (either TAMRA Azide, Biotin Azide, Diazo Biotin Azide, or Dde Biotin Azide, all from Click Chemistry Tools), 6.25 mM tris(2-carboxyethyl)phosphine (TCEP) (Sigma), 0.625 mM tris[(1-benzyl-1*H*-1,2,3-triazol-4-yl)methyl]amine (TBTA) (Sigma), and 6.25 mM CuSO_4_. Appropriate volumes of reagent mix were then delivered to lysates (1:5.25, v/v) to achieve 1% SDS, 100 μM azide, 1 mM TCEP, 100 μM TBTA, and 1 mM CuSO_4_. Typically, 21 μl of lysate was reacted with TAMRA-azide for fluorescence visualization, whereas large-scale reactions with biotin-azide, Diazo biotin-azide, or Dde biotin-azide were conducted using 0.1 to 1.5 ml of lysate. After 1.5 h at RT, TAMRA-labeled samples were prepared for SDS-PAGE as described below. All large-scale reactions with the various biotin-azides were treated with five volumes of ice-cold acetone and allowed to sit at −20 °C overnight. Precipitated proteins were pelleted by centrifugation at 3500*g* at 4 °C for 5 min and washed twice with ice-cold methanol, with 30-min precipitations at −20 °C and centrifugation as above. The final pellets were briefly air dried (<5 min) and then resolubilized according to the relevant protocol for the type of biotin-azide employed.

### OPP-ID Protocol After Click Reaction With Biotin-Azide

#### Affinity Enrichment Using Streptavidin Magnetic Beads

Protein pellets were resuspended in 120 μl of PBS containing 1% SDS and desalted by passing through Zeba spin 0.5 ml 7K cutoff desalting columns (Thermo Scientific) equilibrated with 1% Nonidet P-40, 0.1% SDS in PBS (16). Protein concentrations were measured (BCA assay) and normalized to the lowest concentration by dilution with the elution buffer. Labeled proteins in 100 μl total volume were then immobilized with streptavidin magnetic beads from 90 μl of bead slurry (Pierce, Thermo Scientific) at 4 °C overnight with slow rotation. Beads were washed twice with 0.5 ml of 1% Nonidet P-40, 0.1% SDS in PBS for 10 min, 3 times with 0.5 ml of ice-cold 6 M urea in PBS for 15 min, and 3 times with 0.5 ml of ice-cold PBS for 10 min, all at 4 °C with slow rotation. Finally, beads were rinsed with buffer containing 20 mM Tris-HCl, pH 8.0 and 2 mM CaCl_2_.

#### On-Bead Trypsin Digestion

Proteins immobilized on streptavidin magnetic beads were suspended in trypsinization buffer (20 mM Tris-HCl, pH 8.0 and 2 mM CaCl_2_). Samples were treated with 5 mM DTT at 56 °C for 30 min, followed by alkylation with 20 mM iodoacetamide at RT for 30 min in the dark. Samples were treated once more with 5 mM DTT (5 min), followed by incubation with 500 ng sequencing grade trypsin (Promega) overnight at 37 °C on a shaking incubator. A second portion of 250 ng of trypsin was added in the morning, and the digest was continued for another 4 h. Digestions were stopped by adjusting the solutions to a final concentration of 1% formic acid. The resulting peptides were enriched with C18 Omix Tips (Agilent Technologies) and eluted with 50% acetonitrile containing 0.1% formic acid. The samples were dried by SpeedVac and then resuspended in 0.1% formic acid for analysis by LC-MS/MS.

### Cleavable OPP-ID Protocol After Click Reaction With Dde Biotin-Azide

#### Affinity Enrichment Using Streptavidin Agarose Resin

Protein pellets were first resuspended in an appropriate volume of 1% SDS in PBS containing 10 mM EDTA, such that the amount of EDTA delivered equaled ∼1.2 molar equivalents relative to the quantity of CuSO_4_ present in the initial click reaction mixture. (For reactions conducted at starting protein quantities <500 μg, EDTA concentrations in this solution were reduced to 2.5 or 5.0 mM to allow for the use of workable resolubilization volumes.) This solution was then diluted to get a final concentration of 0.5% SDS in PBS (typical final solution volumes ranged from 150 to 300 μl, depending on the pellet size). Following BCA assay, the protein concentrations were normalized again to give concentrations in the range of 1 to 2 mg/ml. Streptavidin agarose resin (Thermo Scientific) was washed 3 times with PBS and 3 times with 0.5% SDS in PBS, and then a 50% slurry was recreated using 0.5% SDS in PBS. Resin slurry was delivered to the samples (0.2 ml slurry/1 mg of protein), followed by rotation for 1.5 h at RT. Following capture of biotinylated proteins, slurries were transferred to empty spin columns (Bio-Rad mini or micro size, or G-Biosciences <100 μl size, depending on the quantity of resin). Resins were then washed in batch mode with full column volumes (0.2–0.5 ml) of the following solvents with centrifugation at 400 rpm for 1 to 2 min as needed: 10 times with 0.5% SDS in PBS, 6 times with 6 M urea, and 10 times with PBS.

#### Cleavage of the Dde Biotin-Azide Cleavable Linker

Samples immobilized on the streptavidin agarose resin were transferred to Eppendorf tubes and treated with 2% hydrazine in PBS (made from hydrazine monohydrate 64%–65% solution, Sigma-Aldrich) containing 0.05% SDS (from 100- to 250 μl, depending on the volume of resin), with slow rotation for 1 h at RT. Following centrifugation at 1400 rpm for 2 min, the supernatants were carefully recovered. Beads were then treated with a second portion of the 2% hydrazine solution and rotated again for 1 h at RT. Finally, the beads were washed twice with PBS and both supernatants and the washes were pooled and filtered through empty spin columns to remove any residual resin.

#### Concentration and Buffer Exchange of the Released Proteins

To concentrate the released protein solutions, either Microcon YM-3 or Amicon Ultra 0.5 ml 3K filters (Millipore Sigma) were employed. For use with low-level samples, the Amicon Ultra filter inserts were presoaked overnight in 10% EtOH with end-over-end rotation, followed by another 4 to 6 h of rotation in HPLC water, several rinses of the filters with HPLC water, and passage of 0.5 ml of HPLC water through the units immediately before use to remove trace contaminants. Once the initial released protein solutions were concentrated to ∼30 μl, the samples were buffer-exchanged three times with 0.5 ml of 80 mM ammonium bicarbonate and concentrated to a final ∼30 μl. Retentates were carefully recovered with slender pipette tips, and then the filters were rinsed twice with 10 to 20 μl of the buffer. The rinses were collected by inversion of the filters according to the manufacturer’s instructions, and finally, retentates and rinses were pooled. Typically, ∼10% of the solutions were saved for SDS-PAGE.

#### In-Solution Trypsin Digestion

Released proteins in 80 mM ammonium bicarbonate were reduced and alkylated as described above for our on-bead digests and then incubated with 500 ng of sequencing grade trypsin (Promega) overnight at 37 °C on a shaking incubator. In the morning, an additional aliquot of 250 ng of trypsin was added and the incubations were continued for another 4 h. Digestion was stopped by adding 1% formic acid, and the peptides were purified as described above.

### Cleavable OPP-ID Protocol After Click Reaction With Diazo Biotin-Azide

#### Affinity Enrichment Using Streptavidin Agarose Resin

Protein pellets were initially resuspended in 4% SDS containing 10 mM EDTA. As described above, this buffer supplied ∼1.2 molar equivalents of EDTA relative to the original quantity of CuSO_4_ present. Next, this solution was diluted to 0.5% SDS with a buffer containing 1% Brij 97 (Spectrum Chemicals), 150 mM NaCl, and 50 mM triethanolamine, at pH 7.4 (23). Following BCA assay, protein concentrations were normalized to give concentrations in the range of 1 to 2 mg/ml. Streptavidin agarose resin (Thermo Scientific) was washed 3 times with PBS and 3 times with the buffer containing 1% Brij 97, 150 mM NaCl, 50 mM triethanolamine, pH 7.4. A 50% slurry of resin in the last wash buffer was delivered to the samples (0.2 ml slurry/1 mg of protein), followed by rotation for 1.5 h at RT. Following capture of biotinylated proteins, slurries were transferred to empty spin columns as above and resins were then washed in batch mode with full column volumes of the following solvents with centrifugation at 400 rpm for 1 to 2 min as needed (23): twice with 2% SDS in PBS, twice with 8 M urea in 250 mM ammonium bicarbonate, twice with 2.5 M NaCl in PBS, and 2 times each with 0.5, 0.25, and 0.05 M ammonium bicarbonate.

#### Cleavage of the Diazo Biotin-Azide Cleavable Linker

Samples immobilized on the streptavidin agarose resin were transferred to Eppendorf tubes and treated 2 times with 150 μl of 25 mM sodium dithionite in 0.25% SDS for 15 min and 2 times with 150 μl of 25 mM sodium dithionite in 8 M urea for 15 min, at RT with slow rotation (23). Between treatments, the beads were centrifuged at 2500*g* for 2 min to recover the supernatants, which were pooled and filtered through empty spin columns to remove any residual resin.

#### Concentration and Buffer Exchange of the Released Proteins

This step was conducted using Microcon YM-3 filter units (3000 MWCO) as described above for the Dde biotin-azide protocol.

#### In-Solution Trypsin Digestion

Released proteins in 80 mM ammonium bicarbonate were reduced, alkylated, and digested with trypsin, and peptides were purified as described above for the Dde biotin-azide protocol.

### SDS-PAGE

#### In-Gel Fluorescence Visualization

After click conjugation with TAMRA-azide, samples were mixed with 6× Laemmli sample buffer and 15-μL aliquots were resolved by SDS-PAGE using 4 to 12% Bis-Tris, 1.0-mm gels (Life Technologies). Gels were washed briefly with 30% EtOH and water and then scanned for fluorescently labeled proteins and molecular weight markers (All Blue standards; Bio-Rad) using excitation wavelengths of 532 nm and 635 nm, respectively (Typhoon Imaging System; Molecular Dynamics), followed by staining with Coomassie blue reagent.

#### Western Blotting

After click reaction with one of the biotin-azide probes, precipitation, and resolubilization, proteins were resolved by SDS-PAGE as described above, transferred to PVDF membranes, and probed with streptavidin-HRP (Abcam).

#### Silver Staining

Following cleavage of the Dde or Diazo cleavable linker, sample concentration, and buffer exchange, nascent proteins were resolved by SDS-PAGE as described above. Gels were then silver-stained using the Pierce Silver Stain Kit (Thermo Scientific) following the manufacturer’s instructions.

## Mass Spectrometry

### LC-MS/MS Analysis

Peptides resulting from trypsinization were analyzed on a Fusion Lumos Orbitrap mass spectrometer (Thermo Scientific) connected to an M-class NanoAcquity UPLC system (Waters). A 15 cm × 75 μm EasySpray C18 column (Thermo Scientific) was used to resolve peptides using a binary solvent system consisting of 0.1% formic acid in water as mobile phase A and 0.1% formic acid in acetonitrile as mobile phase B. Samples were loaded at 2% B at 600 nl/min for 10 min, and then the system was adjusted to 5% B over 3 min as the flow was reduced to 300 nl/min, followed by a gradient from 5 to 30% B over 72 min, an increase to 70% B over 2 min, a hold at 70% B for 1 min, followed by a decrease to 2% B over 2 min with concomitant increase in flow rate to 600 nl/min, and re-equilibration at 2% B for 6 min. For two sample sets, this standard gradient was modified slightly at the second step to adjust to 3.5% B over either 3 or 5 min, with all other time points remaining unchanged. The instrument was operated in the data-dependent mode to automatically switch between MS and MS/MS. Precursor ions were measured in the Orbitrap scanning from 375 to 1500 *m/z* (full MS resolution, 120,000), and precursor ions with a charge state of 2+ or higher were selected with an isolation window of 1.6 *m/z* and fragmented by HCD in the Orbitrap (resolution, 30,000). A 30-s dynamic exclusion time was used, and the instrument was configured to fragment as many precursors as possible in a 3-s cycle time.

For LC-MS/MS analysis of peptide digests from whole proteome samples, we used a 50 cm x 75 μm EasySpray C18 column (Thermo Scientific) with a flow rate of 300 nl/min and a 245-min run time. Samples were loaded at 3.5% B for 23 min, followed by a gradient from 3.5 to 30% B over 182 min, an increase to 50% B over 2 min, an increase to 70% B over 2 min, a hold at 70% B for 5 min, a decrease to 3.5% B over 2 min, and then re-equilibration at 3.5% B for 29 min. Other instrument settings were as indicated above.

### MS Data Analysis

Peak lists were prepared using PAVA, an in-house program (24). All peak lists from LC-MS/MS runs in each experiment were searched in combination using Protein Prospector (v 6.1.0) against the human entries of a SwissProt database (2017.11.01; 20,240/556,006 entries searched) concatenated with a corresponding random decoy database to allow for false discovery rate (FDR) estimation. The database search was performed with the following parameters: a mass tolerance of 10 ppm for precursor masses and 30 ppm for MS/MS fragment ions, cysteine carbamidomethylation as a fixed modification, and several common variable modifications (including acetylation of protein N termini, methionine loss from protein N termini, oxidation of methionine, and pyroglutamate formation from N-terminal glutamine). Up to two modifications per peptide were allowed and the enzyme was specified as trypsin with two missed cleavages allowed. All searches were performed with a fixed 1% FDR at the protein and peptide levels. These combined searches generated master lists of all identified proteins in each experiment. To report proteins in individual fractions, accession numbers from all identified proteins were supplied to the Search Compare program in Protein Prospector in “Multi Sample” mode. To facilitate protein sequence coverage comparisons, the “Spot/Fraction” option in Search Compare was employed to select biological replicates to be merged, again with the accession numbers from all identified proteins from the combined search supplied.

To search for peptides modified with OPP at the C terminus, additional user-defined modifications were included in the Protein Prospector search parameters. We allowed for no enzyme specificity at peptide C termini and a C-terminal modification of OPP plus a residual C_3_H_8_N_4_ moiety from the cleavable linker, for a total mass modification of C_27_H_35_N_11_O_4_ (577.2873 Da). In addition to intact OPP, we allowed for neutral loss of the purine moiety from OPP (-C_7_H_9_N_5_, -163.0858 Da) with corresponding modification of the C terminus as C_20_H_26_N_6_O_4_ (414.2016 Da). Otherwise, the searches were conducted with the settings noted above. To focus on OPP-modified peptides, peak lists were filtered to include only spectra with the diagnostic fragment ion at *m/z* 164.09 using the “MS-Filter” tool in Protein Prospector.

### Label-Free Quantification

Using Search Compare in Protein Prospector, all accession numbers from proteins identified at a 1% FDR in the combined searches were submitted for MS1 quantification of precursor ions in “Multi Sample” mode. Extracted peptide ion chromatograms were generated using a retention time window of −10/+30 s from when the precursor was selected for MS/MS, and ion intensities for all quantified peptides were reported. Shared peptides in the Protein Prospector output file were assigned uniquely to the highest-ranked protein containing their sequence and after this filtering, all remaining unique peptide entities (in all charge states) were included in the quantitative analysis. Protein intensities were calculated from the sum of the individual unique peptide MS1 precursor ion intensities.

### Filtering of MS Results

Protein identification datasets were assembled including number of unique peptides identified and summed peptide intensity values per protein in all replicates of all conditions for each experiment. For the experiments comparing protocols with PBS-treated and OPP-treated samples, and for the limit of detection experiment using the Dde biotin-azide protocol, all quantified proteins were listed. For the experiment involving inhibition of mTOR with MLN128, the quantified nascent proteins were further filtered to remove any proteins identified in any PBS-treated control sample with >1 peptide and keep only candidates detected in at least two biological replicates in either of the OPP-treated conditions (± MLN128) with ≥2 unique peptides. Protein intensity ratios in the OPP-treated conditions were calculated as MLN128-treated/vehicle-treated. These ratios were converted to log2 values, medians of the three replicates were centered to the global median, and *p*-values were calculated using a two-tailed, one-sample Student’s *t* test against the null hypothesis (assumed mean of 0). For the volcano plot, -log10(*p*-values) were plotted against average log2 fold change (FC), and candidates with |log2 FC| >1 and *p*-values <0.05 met the significance cutoff.

## Bioinformatics

To compare background proteins from PBS-treated control samples with nascent proteins from OPP-treated samples, we used the online Contaminant Repository for Affinity Purification resource (CRAPome v2.0), available at https://reprint-apms.org/. We selected “*H. sapiens*–Single Step Epitope tag AP-MS,” and then provided lists of protein accession numbers for our different protein groups. Our proteins were queried against a total of 716 negative control experiments in the database under this category. Thus, the CRAPome frequency values given in this report represent the proportion of these 716 control experiments in which a given protein was detected as background. In addition to using this database, we also compared the background proteins detected in this study with entries on the common Repository of Adventitious Proteins (cRAP) list (https://www.thegpm.org/crap/).

For assessment of protein abundances in the K562 cell proteome in our limit of detection experiment, we used the PaxDb4.2 Protein Abundance Database (25, 26), available at https://pax-db.org/. We downloaded the dataset for K562 cells derived from the 2012 study of Geiger *et al*. (27), which consisted of 3337 Ensembl Protein IDs with abundance data (9606-K562_Geiger_2012.txt, downloaded 03/28/22). To map protein IDs from Ensembl to UniProt IDs, we used the PaxDb4.2 conversion file available on the website (paxdb-uniprot-links-v4.2.tsv). We found 1989 of our 3431 quantified proteins (58%) in the K562 dataset, and these matched proteins were used in our analyses.

To obtain protein functional classifications, we used the PANTHER Classification System, PANTHER17.0 (http://www.pantherdb.org/) (28). Gene Ontology (GO) networks were classified using the DAVID Bioinformatics Database 2021 (https://david.ncifcrf.gov/), and protein functional interaction networks were visualized using the STRING app in Cytoscape v3.9.1 (https://cytoscape.org/) (29), with the confidence (score) cutoff set to 0.80. Histograms and scatter plots were made using Prism 6 (GraphPad Software) or Microsoft Excel. Proportional Venn diagrams were generated using BioVenn (https://www.biovenn.nl/) (30).

## Experimental Design and Statistical Rationale

Except for our preliminary studies, which were run as biological duplicates, all experiments in this report were run with three biological replicates in all treatment conditions and controls. Controls in OPP treatment experiments involved treatment of cells with PBS (vehicle) instead of OPP. In the mTOR inhibition experiment, controls also included pretreatment of cells with DMSO (vehicle) instead of MLN128. To assess the statistical significance of protein FCs between treatment conditions (± MLN128) in the mTOR inhibition experiment, log2 FC ratios were averaged across the three biological replicates and *p*-values were calculated using a two-tailed, one-sample Student’s *t* test against the null hypothesis (assumed mean of 0).

## Results

### Preliminary Studies Comparing Two Cleavable Biotin-Azides

**Fig. 2:**
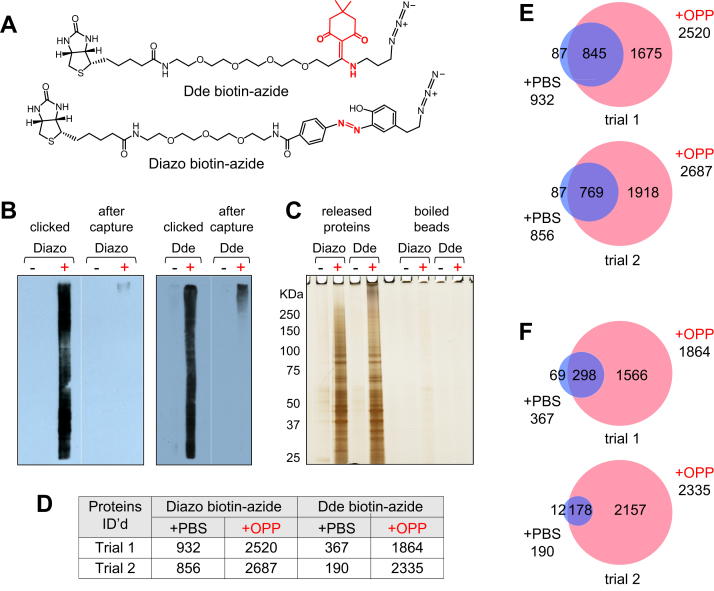
**Comparison of two cleavable biotin-azide linkers.***A*, chemical structures of Dde biotin-azide and Diazo biotin-azide, with the cleavable moieties highlighted in red. *B*, Western blots of clicked samples before and after capture on streptavidin agarose resin, probed with streptavidin-HRP. PBS- and OPP-treated sample lanes are marked with “-” and “+,” respectively. *C*, silver-stained gel of released proteins and material from the residual beads after boiling in Laemmli sample loading buffer. *D*, table summarizing the number of proteins identified in control (PBS-treated) and OPP-treated samples following liquid chromatography/tandem mass spectrometry analysis of released protein digests. *E* and *F*, Venn diagrams showing overlap of proteins detected in the PBS- and OPP-treated samples of the two replicates using (*E*) Diazo-biotin azide and (*F*) Dde-biotin azide. OPP, O-propargyl-puromycin.

To minimize the detection of nonspecifically bound proteins associated with on-bead digestions after affinity enrichment, we sought to incorporate a cleavable biotin-azide linker into our original OPP-ID protocol (16). While several types of cleavable biotin-azides are now commercially available, our preference was to select a chemically cleavable linker. In initial studies, we compared the Diazo biotin-azide and Dde biotin-azide cleavable linkers (Fig. 2*A*) for ease of use and overall performance. For these trials, K562 cells were treated with either PBS (vehicle) or OPP (30 μM) for 2 h to generate the sample sets.

For the Diazo biotin-azide cleavable linker, which is cleaved by mild treatment with 25 mM sodium dithionite (Na_2_S_2_O_4_), we started with an optimized protocol reported by Wilson *et al*. (23). Following OPP labeling and lysis of K562 cells, we performed click chemistry as reported (16), except that Diazo biotin-azide was substituted for biotin-azide in the reagent mix. After reaction, the proteins were acetone precipitated overnight as usual (16). However, for resolubilization of the protein pellets, the specific buffer mixture reported by Wilson *et al*. was employed, which contained EDTA to sequester protein-chelated copper (23). Samples were then captured on streptavidin agarose resin for 1.5 h, the resin was washed in batch mode, and the diazo linker was cleaved using 25 mM sodium dithionite (23). The combined eluates were then concentrated and buffer-exchanged.

In the case of the Dde biotin-azide cleavable linker, which is cleaved with 2% hydrazine (N_2_H_4_), there was some confusion in the literature as to how stable the linker is to primary amines, particularly to Tris buffer in the presence of SDS (31, 32, 33). In devising our protocol for this linker (supplemental Fig. S1), we chose to avoid primary amines but found that low concentrations of SDS alone were not a problem. As with the Diazo biotin-azide, for the Dde biotin-azide linker we performed the click reaction, precipitation, and pellet washing steps using our previous approach. For resolubilization of the protein pellets, we adapted the method described above for the Diazo biotin-azide, using a simpler buffer consisting of only 0.5% SDS in PBS but keeping the EDTA component, which we standardized to ∼1.2 molar equivalents of EDTA relative to the amount of CuSO_4_ present in the click reaction mixture. Samples were then captured on streptavidin agarose resin as above, the resin was washed in batch mode, the Dde linker was cleaved with 2% hydrazine in PBS with 0.05% SDS, and the eluates were concentrated and buffer-exchanged to give the released nascent protein solutions.

We performed two replicates of each of these trial experiments and compared the results. Solutions of biotinylated proteins before and after capture on streptavidin agarose resin were analyzed by SDS-PAGE and probed by Western blotting with streptavidin-HRP. Both experiments showed efficient capture of biotinylated proteins by the resin (Fig. 2*B*). Likewise, SDS-PAGE with silver staining revealed good release of nascent proteins. Both samples showed a ladder of nascent protein bands spanning the molecular weight range (Fig. 2*C*). There was almost nothing detected in the PBS-treated samples or material recovered from the residual beads upon boiling in Laemmli sample loading buffer after the releases were completed (Fig. 2*C*). Following in-solution digestion with trypsin and LC-MS/MS analysis, the numbers of proteins identified in all of the trial samples were tabulated (Fig. 2*D* and supplemental Table S1). As indicated, slightly more proteins were detected in the Diazo biotin-azide experiments than in the Dde biotin-azide experiments. Particularly striking were the low levels of background proteins found in the PBS-treated samples from the Dde biotin-azide protocol as compared with the corresponding samples from the Diazo biotin-azide protocol (Fig. 2*D*). Once overlapping proteins in each pair of PBS- and OPP-treated samples were distinguished (Fig. 2, *E* and *F*), it became clear that the number of proteins that could be characterized as nascent in these two experiments was comparable. However, the dramatically reduced background associated with the Dde biotin-azide cleavable linker made it the superior candidate for further evaluation.

### Direct Comparison of OPP-ID with the Dde Biotin-Azide Cleavable Linker Protocol (OPP-ID_CL_)

For this comparison, K562 cells were treated with either PBS vehicle or OPP (30 μM) for 2 h, with three replicates of each treatment. The six cell pellets were lysed, and their protein concentrations were normalized to 3.5 mg/ml. Small-scale click reactions with TAMRA-azide confirmed good OPP labeling (supplemental Fig. S2, *A* and *B*). Subsequently, 1.2-mg portions of each lysate were taken for the original OPP-ID method and for the Dde biotin-azide cleavable linker protocol, which we have termed OPP-ID_CL_.

The OPP-ID protocol began with an overnight preclear of the samples on streptavidin agarose resin (16), whereas this step was not necessary when using the cleavable Dde biotin-azide because any endogenously biotinylated proteins present in the sample would not end up in the nascent protein pool upon chemical cleavage of the Dde moiety. The click reactions (with either biotin-azide or Dde biotin-azide) and overnight protein precipitation steps were the same for both protocols up to the washings of the protein pellets the next day. From there, the two protocols diverged.

For the samples following the original OPP-ID protocol, pellets were resolubilized in PBS containing 1% SDS, and then the protein solutions were desalted using 7000 MWCO Zeba spin columns before protein concentrations were renormalized. Affinity capture was then performed using streptavidin magnetic beads at 4 °C overnight. Subsequently, the beads were washed and the immobilized samples subjected to on-bead digestion with trypsin (16).

The set of samples following the OPP-ID_CL_ protocol was treated as in our preliminary studies, starting from resolubilization of the protein pellets in EDTA-containing buffer, which removed the need for desalting using the Zeba spin columns described above. After the resolubilization step, we recovered an average of 74% of starting protein quantities using OPP-ID_CL_, compared with a 50% average recovery after desalting using the OPP-ID protocol. For the OPP-ID_CL_ samples, affinity capture was performed on streptavidin agarose resin for 1.5 h at RT, as opposed to the overnight capture on magnetic beads used in OPP-ID. The efficiency of the bead capture step in both protocols was similar, as revealed by SDS-PAGE and Western blot (supplemental Fig. S2, *C* and *D*). After thorough washing of the agarose resin, cleavage of the Dde moiety was conducted using 2% hydrazine in PBS with 0.05% SDS, the eluates were concentrated and buffer-exchanged, and the released proteins were subjected to in-solution digestion with trypsin. All 12 samples from the OPP-ID and OPP-ID_CL_ protocols were analyzed by LC-MS/MS during the same timeframe with identical instrument and HPLC conditions.

**Fig. 3:**
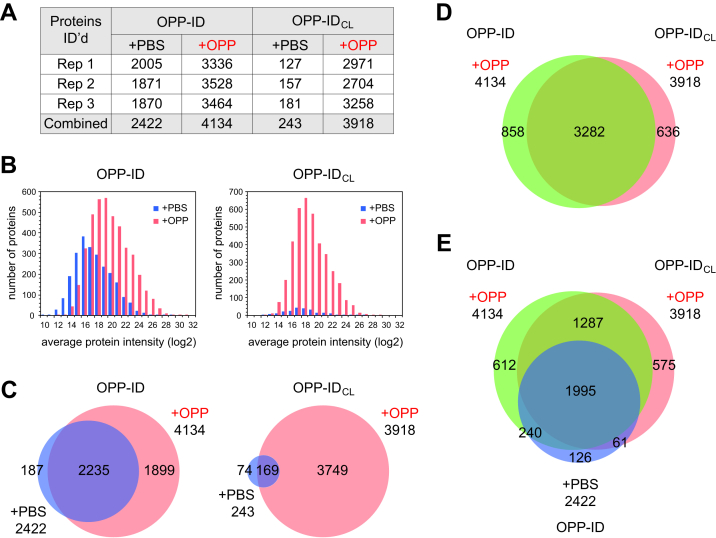
**Comparison of the OPP-ID and OPP-ID**_**CL**_**protocols.***A*, table summarizing the number of proteins identified and quantified in control (PBS-treated) and OPP-treated samples following liquid chromatography/tandem mass spectrometry analysis of protein digests. *B*, binned log2 average protein intensity plots for the OPP-ID and OPP-ID_CL_ protocols. *C*, Venn diagrams showing overlap of combined proteins detected in the PBS- and OPP-treated samples in the two protocols. *D*, Venn diagram showing overlap of proteins in the OPP-treated samples between the two protocols. *E*, Venn diagram indicating additional overlap of the proteins detected in the PBS-treated sample from OPP-ID with both OPP-treated sample sets. OPP, O-propargyl-puromycin.

When searched in combination using Protein Prospector software with a fixed 1% FDR at the peptide and protein levels, 5059 proteins were identified in the full dataset, of which 4936 were quantified (supplemental Table S2). As shown in Figure 3*A*, the combined number of putative nascent proteins identified in the OPP-treated samples was similar in the two experiments, but the number of proteins identified in the corresponding PBS-treated control samples was not. Compared with the original OPP-ID method, there was a 10-fold reduction in the number of background proteins detected in the controls from the OPP-ID_CL_ protocol. Likewise, the overall signal intensity of proteins identified in the OPP-treated samples was similar in the two protocols, but as expected, the protein signal intensity from the PBS-treated control samples was much higher in the OPP-ID than in the OPP-ID_CL_ protocol (Fig. 3*B*).

There was considerable overlap between all proteins detected in the OPP-ID experiment (2235 proteins detected in both the PBS- and OPP-treated samples) (Fig. 3*C*, left). This complicates the designation of such proteins, which are typically removed from consideration as nascent if identified with >1 peptide in any of the PBS-treated controls in biological experiments. In contrast, only 169 proteins fell into this category in the OPP-ID_CL_ experiment (Fig. 3*C*, right). Thus, in this side-by-side comparison, more proteins could be classified as nascent using the OPP-ID_CL_ protocol (3749 proteins) *versus* the original OPP-ID protocol (1899 proteins).

Of all proteins detected in the OPP-treated samples in both experiments, there was 69% overlap (Fig. 3*D*). Concomitantly, the OPP-treated sample from the OPP-ID_CL_ protocol also showed substantial overlap with proteins from the PBS-treated control of the OPP-ID experiment (Fig. 3*E*). To investigate the composition of nascent and background protein groups in these experiments, we turned to the CRAPome to query our protein lists against known background contaminants in affinity purifications. Using the online tool, background proteins detected in 716 relevant experiments were compared with our proteins and the results tabulated by protein accession number (included in supplemental Table S2). From this comparison, we found that both the OPP-ID_CL_ and OPP-ID PBS-treated control (background) samples, 243 and 2422 proteins, respectively, were enriched in abundant CRAPome proteins compared with the nascent protein pool from the OPP-ID_CL_ protocol (3749 proteins) (supplemental Fig. S3*A*). When the nascent proteins in the OPP-ID_CL_ experiment that were not detected in the OPP-ID background were queried independently (1838 proteins), the percentage of proteins found at high levels in the CRAPome was dramatically reduced and the percentage of proteins not detected at all in the CRAPome increased to 18%. In addition to reducing unwanted background, these results highlight another beneficial feature of the OPP-ID_CL_ protocol: true nascent proteins that also have been observed in the CRAPome can be categorized as nascent when appropriate.

**Fig. 4:**
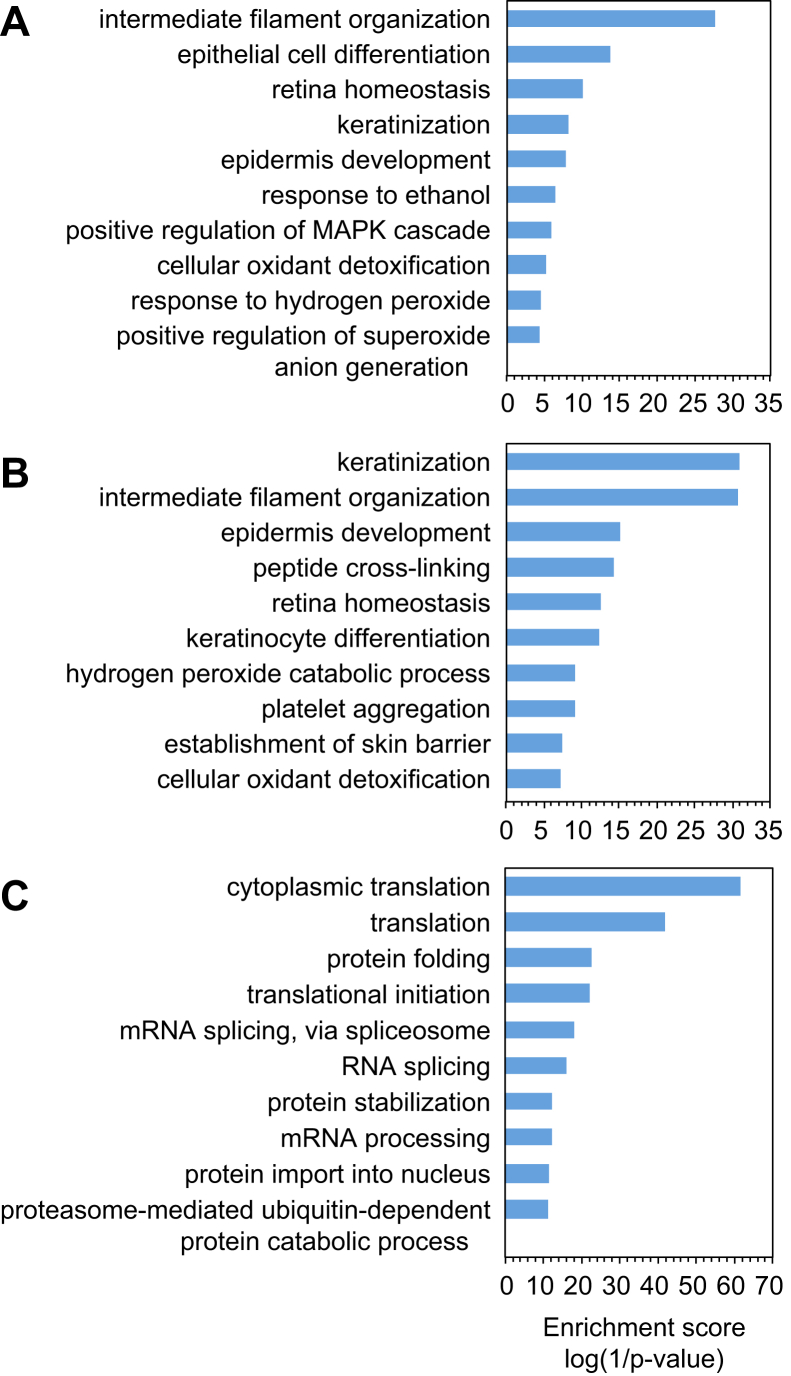
**Comparative Gene Ontology (GO) analysis of background constituents.***A*, GO biological processes associated with the 68 human proteins on the common Repository of Adventitious Proteins (cRAP) list (https://www.thegpm.org/crap/). *B*, GO biological processes enriched in the PBS-treated control samples when using the OPP-ID_CL_ protocol (N = 148 proteins). *C*, GO biological processes enriched in the PBS-treated control samples when using the OPP-ID protocol (N = 1546 proteins). For both protocols, background proteins included in these analyses had ≥2 peptides in at least one biological replicate.

In addition to querying the CRAPome, we also observed that a sizable number of the 243 background proteins found in the PBS-treated controls from the OPP-ID_CL_ protocol were common laboratory contaminants derived from sample handling (*e.g.*, keratins from human skin and hair), many of which are listed in the online cRAP. Gene ontology analysis was performed on a subset of these proteins with ≥2 peptides in at least one biological replicate (148 proteins), as well as on the human cRAP proteins (68 proteins) and the corresponding background from the OPP-ID experiment (1546 proteins). The OPP-ID_CL_ background aligned closely with the human cRAP proteins, enriched in biological processes such as keratinization and intermediate filament organization, whereas the OPP-ID background represented a wide variety of biological processes (Fig. 4). This comparison supported the characterization of the OPP-ID_CL_ background as largely comprising laboratory contaminants rather than general sticky proteins.

To further characterize the 2422 OPP-ID background and 3749 OPP-ID_CL_ nascent proteins, peptides were aligned to protein sequences to derive minimal polypeptide lengths of the corresponding proteins at the moment of OPP labeling, as estimated by the last amino acid residue identified in the set of peptides for a given protein (supplemental Fig. S4*A*). In addition, peptide starting positions (first amino acids) were binned and plotted for the two samples (supplemental Fig. S4, *B* and *C*). Both of these analyses showed that the pool of nascent proteins had a higher proportion of shorter polypeptide chains and N-terminal peptides compared with the pool of background proteins from the PBS-treated control sample, consistent with our expectation that incorporation of OPP leads to premature chain termination. Others have also shown that the detection of nascent proteins is biased towards N-terminal peptides (19).

Finally, we compared the protein sequence coverage of the background proteins and nascent proteins (minus any overlapping background proteins) from both protocols. For this analysis, we searched the three biological replicates from each treatment in combination to obtain merged coverage information for proteins from Protein Prospector. As seen in supplemental Fig. S3*B* for proteins identified with ≥2 peptides in each condition, the mean sequence coverage was larger for background proteins than for nascent proteins in both experiments, as might be expected. For the OPP-ID experiment, this difference between background and nascent proteins was statistically significant (*p*-value <0.0001), whereas the small number of background proteins detected in the OPP-ID_CL_ experiment precluded a statistically significant comparison in that case. More importantly, when protein sequence coverage between the nascent proteins in both experiments was compared, a small (∼3%) but statistically significant improvement in sequence coverage was found when using the OPP-ID_CL_ protocol (*p*-value <0.0001).

### Testing the Limits of Nascent Protein Detection Using OPP-ID_CL_

**Fig. 5:**
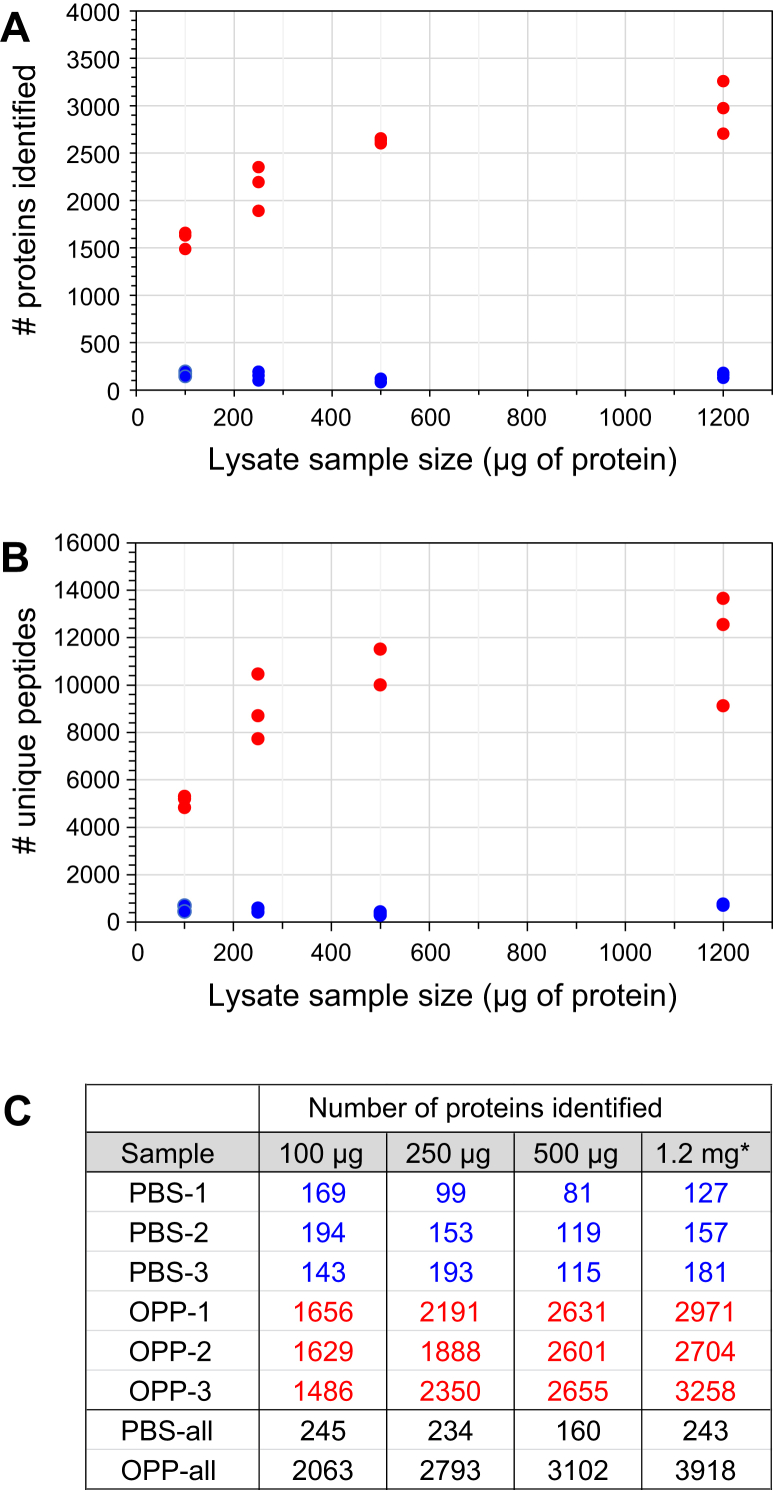
**Performance of the OPP-ID**_**CL**_**protocol with low-level samples.***A*, graph showing the numbers of proteins identified and quantified in the three biological replicates at each sample input size: 100 μg, 250 μg, and 500 μg. *B*, companion graph showing the numbers of unique peptides quantified in the same samples. *C*, table giving the numerical protein counts per fraction, and the overall totals of quantified proteins per condition. In these figures, PBS-treated samples and OPP-treated samples are indicated with blue and red colors, respectively. ∗The values for a 1.2-mg input, shown here for reference, represent the three replicates of the OPP-ID_CL_ protocol from the comparison experiment discussed above (see Fig. 3). OPP, O-propargyl-puromycin.

For this experiment, we prepared three replicates of K562 cells treated with either PBS or OPP and normalized the protein content in the resulting cell lysates. Portions of the samples were then dispensed to give the following sample input levels: 100 μg, 250 μg, and 500 μg of starting protein. These samples were independently taken through the OPP-ID_CL_ protocol, with size-appropriate volumes and devices used. Tryptic digests resulting from the samples were either loaded in their entirety (for the 100 μg and 250 μg samples) or at a level proportional to our typical loading level for larger samples (2/5 of the 500 μg sample) for LC-MS/MS analysis. When searched as a group, 3503 proteins were identified in the full dataset (3431 quantified). The quantified proteins at each sample size in the three replicates were tabulated (supplemental Table S3) and their numbers plotted (Fig. 5). As indicated, the number of putative nascent proteins identified increased consistently as the protein input levels increased from 100 μg to 500 μg. Yet even at the 100 μg input level, ∼1400 to 1600 proteins were identified in each replicate. Furthermore, the PBS-treated controls from all input levels had <200 background proteins detected.

As shown in the Venn diagrams in supplemental Fig. S5*A*, we examined the overlap of proteins detected in the corresponding PBS- and OPP-treated samples for the three submilligram samples. While all of the samples had quite low levels of background proteins, the proportion of overlapping proteins between the PBS- and OPP-treated samples increased slightly as the sample input size decreased. Next, we compared assigned nascent proteins (minus overlapping background proteins) at the three sample input levels (supplemental Fig. S5*B*). Of the cumulative number of nascent proteins identified and quantified, there was 49% overlap of proteins at all three sample input levels (1605 proteins). When only the subset of nascent proteins identified and quantified with ≥2 peptides per protein in at least one biological replicate was considered (supplemental Fig. S5*C*), there was still 40% overlap of proteins at all sample input levels (851 proteins). Moreover, the 851 common proteins included 89% of all proteins detected in the 100-μg sample with ≥2 peptides, giving us a large subset of well-represented nascent proteins detected in all of the samples. Proteins from the PBS-treated controls at the three sample input levels were also plotted in the same fashion (supplemental Fig. S5, *D* and *E*), showing a 32% overlap of background proteins with ≥2 peptides.

To get a measure of how protein sequence coverage correlated with sample input size, we searched the three biological replicates from each condition of each sample size in combination to obtain merged coverage information from Protein Prospector for these communal proteins. First including all identified proteins and then including only proteins identified with ≥2 peptides, we obtained the scatter plots shown in supplemental Fig. S6, *A* and *B*, respectively. In both cases, the mean protein sequence coverage for the 100-μg sample was significantly lower than for the other two samples (*p*-value <0.0001). However, the mean protein sequence coverage for the 250 μg and 500 μg samples was not distinguishable for these overlapping proteins in either case. This observation suggests that the proportion of final peptide digests taken for LC-MS/MS analysis of the 250 μg and 500 μg samples (100% and 40%, respectively) resulted in comparable quality MS results in those cases, whereas loading 100% of the 100 μg sample gave reduced sequence coverage overall.

To assess the cellular abundance of nascent proteins detected at the various sample input sizes in our experiment, we used the online Protein Abundance Database (PaxDb), where published MS-based protein abundance data are converted to standardized “ppm” values (25, 26). From the relevant PaxDb file for K562 cells, we extracted ppm values for our proteins and then compared the abundances of proteins detected at the three sample input levels (included in supplemental Table S3). As shown in supplemental Fig. S7, we observed statistically significant decreases in mean protein abundance going from smaller to larger starting sample input sizes, although we were able to detect nascent proteins over the same abundance range in all samples. This analysis also revealed that the mean protein abundance of the background proteins was considerably higher than that of any of the nascent protein samples (supplemental Fig. S7*B*).

### MS Detection of C-Terminally Modified Peptides Bearing OPP plus Residual Linker Fragment

Metabolic labeling with OPP can theoretically occur at any point during protein synthesis, resulting in an ensemble of truncated polypeptides representing a given nascent protein. Thus, we assumed that the prospect of detecting appreciable levels of a given C-terminally modified peptide bearing OPP by mass spectrometry was remote. In the studies discussed above, we searched only for canonical peptides derived from the nascent proteins following tryptic digestion.

**Fig. 6:**
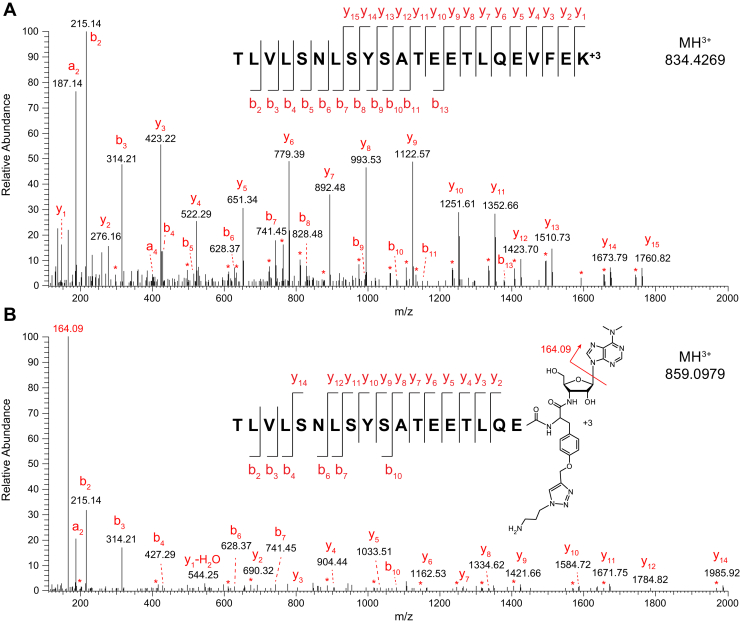
**Identification of a peptide modified with OPP at the C terminus.***A*, tandem mass spectrum of the canonical tryptic peptide TLVLSNLSYSATEETLQEVFEK (MH^3+^ at 834.4269) from Nucleolin (NCL). *B*, tandem mass spectrum of a truncated form of this peptide (MH^3+^ at 859.0979) modified with OPP at the C terminus. Peaks marked with asterisks represent losses of H_2_O or NH_3_ from the main sequence ions. OPP, O-propargyl-puromycin.

When analyzed by tandem mass spectrometry, OPP readily loses its purine moiety from the molecular ion and gives a fragment for the moiety at *m/z* 164 (34). Using a pair of samples prepared from K562 cells treated with either PBS or OPP and processed using OPP-ID_CL_, we found that this diagnostic fragment at *m/z* 164.09 was indeed present in our OPP-treated samples and absent in our PBS-treated controls (supplemental Fig. S8). To enable Protein Prospector to identify and sequence peptides modified with OPP plus a residual C_3_H_8_N_4_ fragment from the cleavable linker, we allowed for no enzyme specificity at the peptide C terminus and added the necessary user-defined modifications for OPP+C_3_H_8_N_4_ (C_27_H_35_N_11_O_4_) at the C terminus plus the option for a neutral loss of the labile purine moiety from the molecular ion (loss of C_7_H_9_N_5_). In examining the results (supplemental Table S4), we found that the best-scoring spectra for modified peptides consistently contained *m/z* 164.09 (C_7_H_10_N_5_^+^) as the base peak. As shown for peptides from Nucleolin (NCL), the C-terminally modified peptides and their corresponding canonical tryptic peptides when present exhibited a shared series of b-ions (Fig. 6). From a single OPP-treated sample, we found 27 reliable identifications of C-terminally modified peptides from a variety of nascent proteins (Table 1). Furthermore, this modification was not detected in any peptides from the PBS-treated sample (supplemental Table S4). The position of OPP incorporation was noted (“Next AA” in Table 1), as well as the corresponding DNA codon and its frequency in the human genome. In three cases, we found OPP incorporated at the protein C terminus, but no other obvious trends were detected in this small dataset.

**Table 1:** Peptides modified with C-terminal OPP^a^

Acc #	Gene	Protein name	*m/z*	z	C-terminally modified peptide	Next AA	Next Codon	Codon FPT
P02545	LMNA	Prelamin-A/C	696.3437	3	NSNLVGAAHEELQQ	S	TCG	4.03
P04908	HIST1H2AB	Histone H2A type 1 B/E	653.3621	3	VTIAQGGVLPNIQA	V	GTG	25.87
P07992	ERCC1	DNA excision repair protein ERCC-1	1403.6621	4	STQSLPTVDTSAQAAPQTYAEYAISQPLEGAGATCPTGSEPLAGETPNQA	L	CTG	36.1
P09972	ALDOC	Fructose-bisphosphate aldolase C	898.7755	3	GVVPLAGTDGETTTQGLDGLSE	R	CGC	8.71
P11021	HSPA5	78-kDa glucose-regulated protein	1240.9471	3	GINPDEAVAYGAAVQAGVLSGDQDTGDLVLLD	V	GTA	7.66
P11142	HSPA8	Heat shock cognate 71-kDa protein	1020.0259	4	SINPDEAVAYGAAVQAAILSGDKSENVQDLLLLD	V	GTC	13.44
P14635	CCNB1	G2/mitotic-specific cyclin-B1	1149.5383	4	LSPEPILVDTASPSPMETSGCAPAEEDLCQAFSDVILA	V	GTA	7.66
P19338	NCL	Nucleolin	859.0979	3	TLVLSNLSYSATEETLQE	V	GTA	7.66
P23193	TCEA1	Transcription elongation factor A protein 1	1145.8602	3	TGDDYIAIGADEEELGSQIEEAIYQE	I	ATA	8.08
P41208	CETN2	Centrin-2	699.3281	3	ELGENLTDEELQE	M	ATG	21.53
P49006	MARCKSL1	MARCKS-related protein	1051.4657	4	GAEASAASEEEAGPQATEPSTPSGPESGPTPASAEQNE	-	TAG	0.35
P52597	HNRNPF	Heterogeneous nuclear ribonucleoprotein F	773.051	3	ITGEAFVQFASQELAE	K	AAG	31.77
P55884	EIF3B	Eukaryotic translation initiation factor 3 subunit B	1082.2559	4	TEPAAEAEAASGPSESPSPPAAEELPGSHAEPPVPAQGE	A	GCC	25.84
P57086	SCAND1	SCAN domain-containing protein 1	903.149	3	AAASAALELPLGPAPVSVAPQAE	A	GCT	18.99
P68363	TUBA1B	Tubulin alpha-1B chain	1106.2401	3	LEFSIYPAPQVSTAVVEPYNSILTT	H	CAC	14.65
Q07021	C1QBP	Complement component 1 Q subcomponent-binding protein, mitochondrial	724.3483	4	GVDNTFADELVELSTALEHQE	Y	TAC	13.49
Q13066	GAGE2B	G antigen 2 B/2C	738.932	5	QDPAAAQEGEDEGASAGQGPKPEAHSQEQGH	P	CCA	18.92
Q13765	NACA	Nascent polypeptide-associated complex subunit alpha	960.129	3	VQGEAVSNIQENTQTPTVQEE	S	AGT	14.05
Q13765	NACA	Nascent polypeptide-associated complex subunit alpha	752.6938	3	NNSNDIVNAIMELTM	-	TAA	0.44
Q14103	HNRNPD	Heterogeneous nuclear ribonucleoprotein D0	794.3727	3	EYFGGFGEVESIELPM	D	GAC	24.27
Q14318	FKBP8	Peptidyl-prolyl cis-trans isomerase FKBP8	1057.5203	3	EFLAAMEPEPAPAPAPEEWLDILG	N	AAC	18.3
Q14974	KPNB1	Importin subunit beta-1	1005.4691	3	ESTLEAIGYICQDIDPEQLQD	K	AAA	27.48
Q15942	ZYX	Zyxin	765.7106	3	EVEELEQLTQQLMQ	D	GAC	24.27
Q8NC51	SERBP1	Plasminogen activator inhibitor 1 RNA-binding protein	779.7021	3	SSASAPDVDDPEAFPALA	-	TAA	0.44
Q92804	TAF15	TATA-binding protein-associated factor 2N	969.0908	3	TDADSESDNSDNNTIFVQGLGE	G	GGT	10.83
Q99729	HNRNPAB	Heterogeneous nuclear ribonucleoprotein A/B	817.7206	3	EYFGEFGEIEAIELPM	D	GAT	24.03
Q9UEU5	GAGE2D	G antigen 2D	799.7582	5	QDPAAAQEGEDEGASAGQGPKPEADSQEQGHPQT	G	GGG	15.35

a Peptides were identified with OPP plus a residual C_3_H_8_N_4_ moiety from the cleavable linker on their C termini, and their MS/MS spectra contained the diagnostic fragment ion at *m/z* 164.09. The position of OPP incorporation is indicated as the “Next AA,” alongside the corresponding DNA codon. The codon frequency per thousand (FPT) values were obtained from the FDA website, https://dnahive.fda.gov/dna.cgi?cmd=codon_usage&id=537&mode=cocoputs.

### Nascent Proteome Upon Inhibition of mTOR

The mTOR signaling pathway regulates global protein synthesis and cell growth in response to nutrients and other cues (35, 36). In our earlier report (16), we tested the ability of the OPP-ID method to detect changes in protein expression resulting from pretreatment of K562 cells with MLN128, a second generation, ATP-competitive active-site inhibitor of the mTOR kinase (35). In that study, we identified a set of 217 nascent proteins, of which 52 showed strong downregulation (log2 FC <-1) upon inhibitor treatment, including 28 with *p*-values <0.05 (16). Here, we performed a similar experiment with K562 cells using OPP-ID_CL_ to see if incorporation of the cleavable linker would allow for detection of a larger group of differentially expressed nascent proteins and enhance our understanding of mTOR-dependent downstream effects.

For this experiment, K562 cells were first treated with either DMSO or 300 nM MLN128 for 1 h, followed by the addition of either PBS or OPP for 2 h. Three biological replicates of the experiment were conducted, and incorporation of OPP was confirmed by small-scale click reactions with TAMRA-azide (supplemental Fig. S9, *A* and *B*). Samples (1.75 mg) were then taken through the OPP-ID_CL_ protocol, followed by LC-MS/MS analysis.

**Fig. 7:**
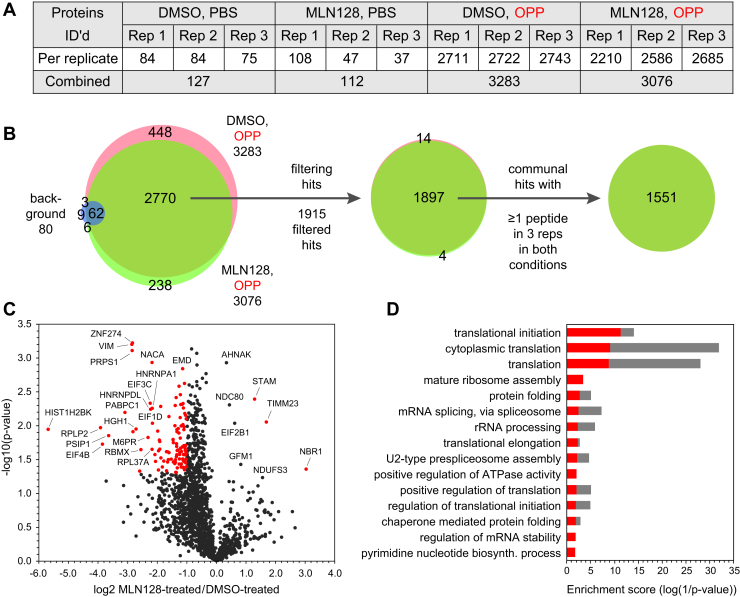
**Treatment of K562 cells with an inhibitor of the mTOR pathway.***A*, summary table of quantified proteins in the three biological replicates. *B*, Venn diagrams showing the overlap of nascent proteins in the two O-propargyl-puromycin (OPP)-treated sample conditions ( ± MLN128) including overlap of the 80 background proteins filtered out of the full dataset (*left*), the overlap of the 1915 hits remaining after filtering with our inclusion/exclusion criteria (*middle*), and extraction of 1551 communal hits with no missing values (*right*). *C*, volcano plot of the 1551 communal hits, showing -log10(*p*-value) *versus* protein intensity fold change (log2) in MLN128-treated/dimethyl sulfoxide (DMSO)-treated cells. *Red dots* represent statistically significant hits with |log2 FC| >1 and *p*-values <0.05. *D*, Gene Ontology biological processes enriched in the subset of proteins with log2 FC <-1 and *p*-values <0.05 (*red bars*) and corresponding enrichments in all proteins with log2 FC <-1 (*gray bars*).

A total of 3629 proteins were identified in the full dataset (1% FDR at the peptide and protein levels), and results following quantification are given in supplemental Table S5 and summarized in Figure 7*A* (3547 proteins quantified). As in our other experiments using OPP-ID_CL_, the levels of background proteins in the PBS-treated controls were quite low. For the pairs of OPP-treated samples ( ± MLN128), there were consistently more proteins identified in the DMSO-treated *versus* the MLN128-treated samples, although the numbers were not too disparate. There was good overlap between quantified proteins in the three replicates of both OPP-treated conditions (supplemental Fig. S9, *C* and *D*). Likewise, when the OPP-treated replicates were combined, there was 80% overlap of proteins in the DMSO- and MLN128-treated samples (Fig. 7*B*).

From this point, the full dataset was filtered to exclude all proteins detected in any control condition with >1 peptide and include only proteins detected with ≥2 peptides in at least two biological replicates of one of the OPP-treated conditions ( ± MLN128) (supplemental Table S5). This resulted in 1915 filtered hits (Fig. 7*B*), for which FC ratios were calculated per replicate (as MLN128-treated/DMSO-treated) and converted to log2 values. The log2 FC median of each replicate was centered to the global median prior to averaging. This new, nearly 9 times larger set of filtered hits included 199 of the 217 filtered hits from our earlier report (16).

For the 1551 hits detected in all samples with no missing values (Fig. 7*B*), *p*-values were calculated and plotted against average log2 FC for MLN128-treated/DMSO-treated samples (Fig. 7*C*). As indicated by the asymmetry of the volcano plot, the MLN128 treatment led to broad repression of global protein synthesis. A total of 110 proteins were categorized as significantly changed (with |log2 FC| >1 and *p*-values <0.05), with 107 proteins downregulated and 3 proteins upregulated as a result of the MLN128 treatment.

The 107 downregulated proteins were analyzed using the DAVID bioinformatics database. As shown in Figure 7*D*, there was strong enrichment for GO biological processes including translational initiation, cytoplasmic translation, and translation, consistent with our previous findings (16). When all 519 downregulated proteins with log2 FC <-1 were included in the analysis (supplemental Table S5), these results were reinforced, leading to very strong enrichment scores (Fig. 7*D*).

**Fig. 8:**
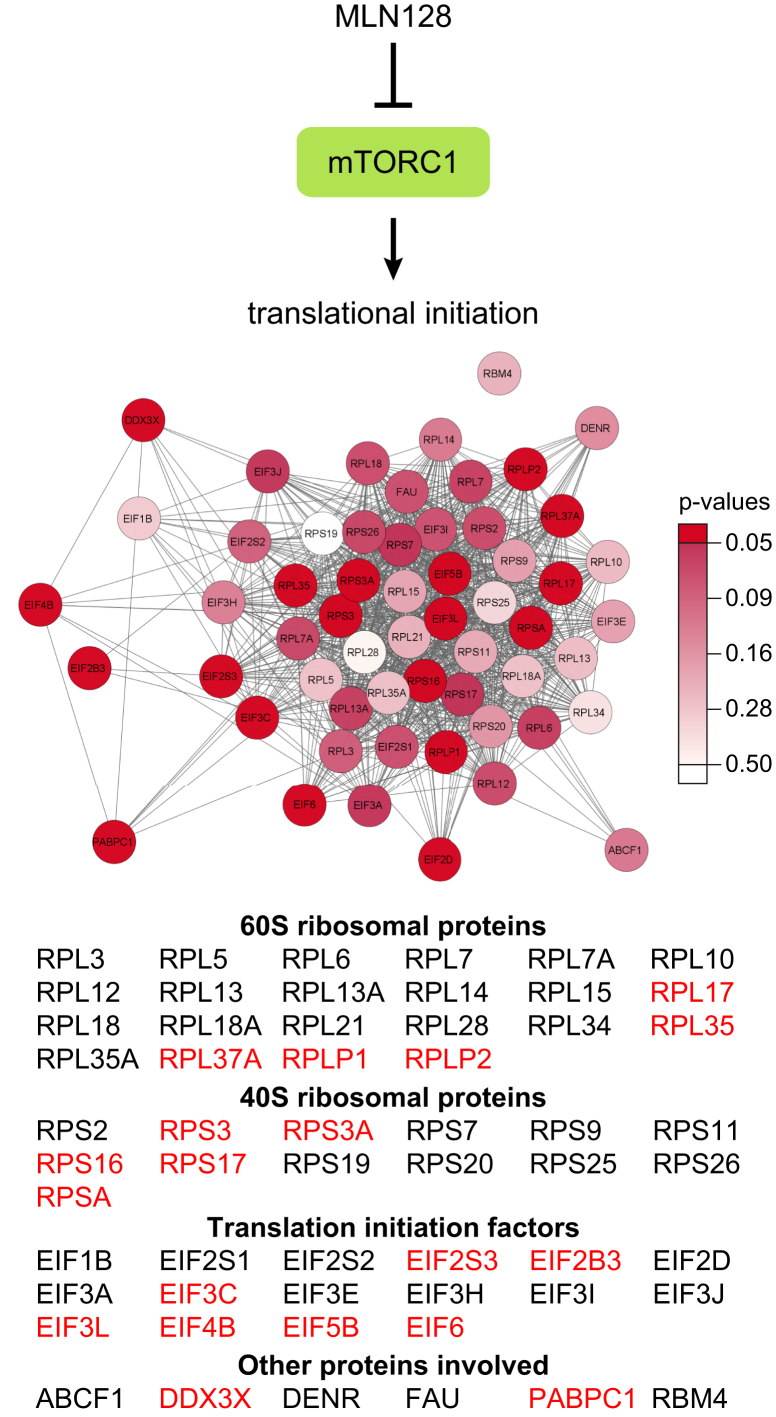
**Subnetwork of downregulated proteins involved in translational initiation.** This functional network was extracted from the full network of downregulated proteins shown in supplemental Fig. S10. The diagram is color coded by *p*-values, with *dark red* indicating *p*-values <0.05, the gradient of lighter shades reflecting higher *p*-values, and white indicating no *p*-value available. Gene names given in red font represent significantly downregulated proteins with log2 FC <-1 and *p*-values <0.05.

Using the STRING app in Cytoscape, a network of 351 functionally interacting proteins was generated with high confidence from the 519 proteins repressed by MLN128 treatment (supplemental Fig. S10). The major feature of this network was a tight cluster of 57 proteins involved in translational initiation (FDR = 1.82E-39), which included 19 of our significantly downregulated hits. The group included various translation initiation factors and numerous 40S and 60S ribosomal proteins (Fig. 8 and supplemental Table S6). One of the most highly downregulated proteins, Eukaryotic translation initiation factor 4B (EIF4B), is a positive regulator of 5′ cap-dependent translation (36), and of our 57 proteins involved in translational initiation, 49 are associated with the Reactome Pathway for cap-dependent translation initiation (supplemental Table S6).

Other major clusters in the network of downregulated proteins contained proteins involved in oxidative phosphorylation (linked to the citric acid cycle and respiratory electron transport pathway) and mRNA splicing, via spliceosome (processing of capped intron-containing pre-mRNA) (supplemental Fig. S10 and supplemental Table S6). Smaller groups of proteins were associated with cell cycle process, cholesterol biosynthesis, vesicle-mediated transport, and iron–sulfur cluster assembly. In addition to these prominent network clusters, many other metabolic processes were associated with smaller protein groupings in the network, reflecting a myriad of downstream effects resulting from MLN128 inhibition of the mTOR signaling pathway.

**Fig. 9:**
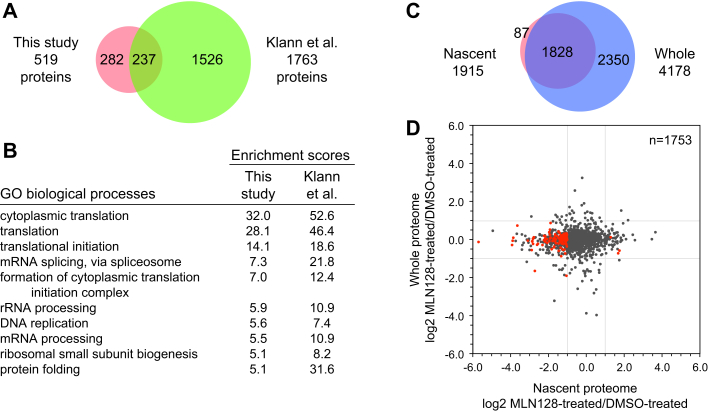
**Comparative analyses of mTOR inhibition.***A*, overlap between the 519 downregulated proteins detected in this study and 1763 proteins from the study of Klann *et al*. (10) also downregulated with log2 FC <-1. *B*, the *top* 10 Gene Ontology biological processes repressed in our study were also enriched in the dataset of Klann *et al*. *C*, overlap of filtered hits from our analyses of nascent and whole cell proteomes in K562 cells. *D*, scatter plot correlating log2 FC values of proteins seen in both the nascent and whole cell proteomes.

While investigating regulation of translation during stress, Klann *et al*. recently discovered a set of proteins regulated by both the integrated stress response and mTORC1 pathways (10). As part of their studies, they probed the nascent proteome upon mTOR inhibition for 9 h with Torin-1 in HeLa cells using mePROD proteomics. Similar to our observations, Klann *et al*. also observed strong inhibition of global translation upon mTOR inhibition. To facilitate a direct comparison of our results, we compared our 519 downregulated proteins with the 1763 proteins downregulated with log2 FC <-1 identified by Klann *et al*. (10). As shown in Figure 9*A*, 46% of our hits were also found in the Klann *et al*. dataset. More importantly, when analyzed using the DAVID bioinformatics database, there was excellent agreement between the significantly repressed biological processes in our two datasets, with cytoplasmic translation as the top GO term in both cases (Fig. 9*B*).

In contrast to the extensive network of repressed nascent proteins observed in our study, only three proteins, Next to BRAC1 gene 1 protein (NBR1), Mitochondrial import inner membrane translocase subunit Tim23 (TIMM23), and Signal transducing adapter molecule (STAM), were detected as significantly upregulated upon MLN128 treatment (Fig. 7*C*). When combined with all 84 upregulated proteins with log2 FC >1 (supplemental Table S5), there was very modest enrichment for GO biological processes including cellular response to calcium ion, blastocyst hatching, and endoplasmic reticulum to Golgi vesicle-mediated transport (supplemental Fig. S11). These processes are clearly distinct from the GO biological processes associated with the downregulated proteins (Fig. 7*D*).

The 84 upregulated proteins did not form a coherent network when examined using the STRING app in Cytoscape (data not shown). When classified using the PANTHER protein classification system, the upregulated proteins fell into 15 general functional categories, with the top categories being metabolite interconversion enzymes, RNA metabolism proteins, and protein-modifying enzymes (supplemental Fig. S12*B*). These 15 functional categories were also represented in the 519 downregulated proteins, as well as 5 additional categories unique to that set (supplemental Fig. S12*A*). The most significant difference between the two groups was the large proportion of translational proteins present in the set of downregulated proteins (supplemental Fig. S12), again highlighting the main effect of mTOR inhibition by MLN128.

In a parallel experiment, we analyzed the whole cellular proteomes from the OPP-treated samples used to characterize the nascent proteome upon acute mTOR inhibition with MLN128 (samples treated with either DMSO or MLN128 for 1 h prior to OPP treatment). A total of 5870 proteins were identified in the full dataset (1% FDR at the peptide and protein levels), with 5713 proteins quantified (supplemental Fig. S13*A* and supplemental Table S7). Upon filtering for proteins with ≥2 peptides in ≥2 biological replicates in either treatment condition, 4178 proteins met our criteria for inclusion (supplemental Fig. S13*B*). The 3898 hits with no missing values were plotted and showed a symmetrical volcano plot (supplemental Fig. S13*C*), in contrast to the asymmetrical plot characteristic of the nascent proteome (Fig. 7*C*). When the small numbers of differentially expressed proteins were analyzed using the DAVID bioinformatics database (294 downregulated proteins with log2 FC <-1 and 288 upregulated proteins with log2 FC >1), only very modest enrichments in GO biological processes were observed. The top enriched categories (supplemental Fig. S13, *D* and *E*) did not correlate well with the enriched biological processes associated with the nascent proteome (Figs. 7*D* and S11).

Of our 1915 filtered hits in the nascent proteome (Fig. 7*B*), 1828 were detected in the corresponding whole proteome (Fig. 9*C*). Log2 FC values for proteins measured in two or three biological replicates in both the nascent and whole proteomes were plotted to compare the enrichments in the two sample sets (Fig. 9*D*). As is shown in the plot, with only a few exceptions, the proteins strongly downregulated in the nascent proteome did not show a similar trend in the whole proteome. Clearly, proteomic changes measured in the nascent proteome were not detectable in the whole proteome samples.

## Discussion

We have optimized the selectivity and performance of our original OPP-ID method for capturing and identifying nascent proteins (16) by incorporating a cleavable biotin-azide linker into the protocol. In side-by-side experiments, we demonstrated improved sample recovery after click reaction, a dramatic reduction in background proteins (nonspecific binders) detected in PBS-treated controls, and more assigned nascent proteins when using OPP-ID_CL_ (Fig. 3). In addition, a modest but statistically significant increase in nascent protein sequence coverage was realized when using OPP-ID_CL_ *versus* original OPP-ID (supplemental Fig. S3*B*). Even at the lowest sample input level tested (100 μg starting protein), we were still able to detect ∼1400 to 1600 nascent proteins, maintain low background protein levels, and detect proteins over the full abundance range represented in the PaxDb K562 dataset (ppm values spanning six orders of magnitude) (Figs. 5 and S7).

Fundamentally, these improvements stem from the substitution of an in-solution digestion of released nascent proteins for the on-bead digestion used in the original protocol, where nonspecific binders reduce the specificity of the results. Even with stringent washing conditions, affinity purifications that rely on on-bead digestions always contain CRAPome proteins (18). In this regard, the advantage of the OPP-ID_CL_ method is twofold: sticky proteins bound nonspecifically to the streptavidin beads remain behind upon cleavage of the cleavable linker, whereas true nascent proteins are selectively released, including any nascent proportion of would-be sticky proteins that otherwise might be disregarded if also detected in background. Thus, when using OPP-ID_CL_, we get a more accurate view of the full range of nascent proteins undergoing elongation during the OPP incubation period. Furthermore, we found that the small OPP-ID_CL_ background is skewed toward keratins and other contaminants introduced by sample handling, rather than toward the general population of sticky proteins (Fig. 4).

As a method to measure nascent proteomes, OPP labeling is a simple approach that does not involve the depletion of endogenous amino acids associated with SILAC or noncanonical amino acid labeling. AHA or HPG incorporation cannot label ∼6% of the human proteome (proteins lacking methionine or proteins with a single N-terminal methionine that is easily removed posttranslationally) (37), whereas OPP can label any nascent chain and access the whole proteome. One disadvantage is that only a single OPP label is incorporated per polypeptide chain and unlike peptides labeled with noncanonical amino acids, this C-terminally modified peptide is typically not detected in digests analyzed by mass spectrometry. As another limitation, the chain-terminating aspect of OPP labeling, with subsequent release of truncated polypeptides and disassociation of the ribosome, could potentially affect cellular processes under investigation, although the use of 30 μM OPP for nascent proteome labeling was previously shown to have no effect on cell viability or markers of cellular stress (16). In AHA and HPG labeling, the media adjustments and concentrations of noncanonical amino acids used to overcome incorporation rates 400 and 500 times lower than methionine, respectively (3), could potentially also affect cellular processes. As for all nascent proteomics workflows, proper controls are needed to control for any perturbations associated with the labeling methods.

Despite the stochastic nature of OPP incorporation into elongating polypeptide chains, which results in limited accumulation of individual species, we were able to identify a small number of peptides modified with OPP at the C terminus by mass spectrometry when we attempted to search for them, providing experimental evidence of the expected covalent modification. As noted in a recent review (14), it has been proposed that puromycylation may occur more readily at times of ribosome pausing (on rare codons, for example), which leaves the ribosome A-site unoccupied longer than usual. Consistent incorporation of OPP at sites of ribosome pausing may generate sufficient quantities of specific OPP-tagged polypeptides such that MS detection of their C-terminally modified peptides is possible following tryptic digestion. Furthermore, it has been reported that, at very low concentrations (0.04–1.0 μM), puromycin cannot compete effectively with aminoacyl-tRNAs and thus only gets incorporated when ribosomes reach a stop codon (38), which leads to full-length puromycylated proteins. During our 2-h incubation period, OPP is progressively depleted from the media, potentially reaching such low micromolar concentrations. Indeed, in our small set of C-terminally modified peptides from a single lysate (Table 1), we detected three OPP-modified peptides representing protein C termini. While by no means comprehensive, our successful detection of puromycylated peptides by mass spectrometry demonstrates the feasibility of investigating OPP incorporation directly.

The K562 cell line used in our experiments is a chronic myeloid leukemia model system, and as in many human cancers, dysregulation of the mTOR pathway is associated with disease progression (39). Here, we treated K562 cells with MLN128, a second-generation ATP-competitive mTOR inhibitor capable of inhibiting phosphorylation of all substrates of mTOR in both protein complexes, mTORC1 and mTORC2 (35, 40). As in our earlier work using OPP-ID (16), acute treatment with MLN128 resulted in strong repression of global protein synthesis, but here when using OPP-ID_CL_, we obtained a considerably richer network of 519 downregulated nascent proteins with log2 FC <-1 (supplemental Fig. S10), including 107 with *p*-values <0.05. By far, the most significant group of downregulated proteins was involved in translational initiation, a process mediated by mTOR in mTORC1 (36). These 57 repressed proteins included both translation initiation factors and structural components of the ribosome (Fig. 8 and supplemental Table S6). Two of the most highly downregulated proteins detected in this study, Eukaryotic translation initiation factor 4B (EIF4B) and Polyadenylate-binding protein 1 (PABPC1) (Fig. 7*C*), are involved in binding to the 5′ end of mRNA, facilitating the recruitment of activated mRNP to the preinitiation complex (41). Repression of these proteins inhibits a downstream program of 5′ cap-dependent translation that affects global protein synthesis (36, 42). In a previous study using ribosome profiling in mouse embryonic fibroblasts treated with the ATP-competitive mTOR inhibitor Torin-1, translation of *Eif4b* was found to be suppressed, in addition to mRNA translation of nearly all cytoplasmic ribosomal proteins (42). Here, we have obtained protein-level evidence for this translational program repressed by an ATP-competitive mTOR inhibitor, consistent with the recent report of Klann *et al*. (10), which identified a large group of proteins repressed by 9-h treatment of HeLa cells with Torin-1 that partially overlapped with our downregulated proteins (Fig. 9*A*) and showed strong agreement in enriched GO biological processes (Fig. 9*B*). While smaller clusters of proteins involved in other metabolic processes such as mRNA splicing via spliceosome and oxidative phosphorylation were also downregulated in our study (supplemental Fig. S10 and supplemental Table S6), the overriding effect of MLN128 treatment of K562 cells was strong repression of components of the translational machinery.

The most highly downregulated protein observed in this study, Histone H2B type 1-K (HIST1H2BK) (Fig. 7*C*), is among a group of proteins involved in chromosome organization. In a recent report, RNA-binding proteins (RBPs) were identified as critical regulators of myeloid leukemia, with extensive studies focusing on the RBP staufen 2 (Stau2) (43). This RBP was shown to be a regulator of a group of chromatin modifiers that included HIST1H2BK, and knockdown of HIST1H2BK significantly reduced the colony-forming ability of K562 cells, leading to speculation that it may be a newly discovered driver of leukemia propagation (43). Considering these findings, our detection of HIST1H2BK as very highly downregulated upon MLN128 treatment of K562 cells is intriguing and may warrant further investigation of this leukemia-associated target.

Inhibition of mTOR is expected to have two parallel effects: downregulation of global translation and restoration of autophagy-related processes. While our dataset consisted primarily of downregulated proteins, there were a few proteins found to be significantly upregulated upon MLN128 treatment: NBR1, TIMM23, and STAM. NBR1 is known to play a protective role in facilitating the autophagic degradation of misfolded or mutated proteins as a selective autophagy cargo transporter (44, 45). In a recent proteomic study of mTOR-regulated autophagy in mouse embryonic fibroblasts, upon 17-h treatment with MLN128 in glucose-containing media, NBR1 was among the autophagy receptors found to be elevated in a knockout lacking the known autophagy gene *atg5 versus* wildtype (46). Expression of the NBR1 gene was also found to be highly upregulated upon either nutrient deprivation or 8-h treatment with the ATP-competitive mTOR inhibitor AZD8055 in HEK293 or SHSY-5Y cells, leading to speculation that its promoter is heavily regulated under these conditions (44). In an *in vitro* study of urothelial carcinoma of the bladder involving treatment with rapamycin, the NBR1 gene was also found to be upregulated, and in the same report, clinical samples linked upregulation of NBR1 with higher recurrence of the disease (47). The authors also showed that knocking out the NBR1 gene potentiated rapamycin-induced inhibition of cell growth and proliferation in urothelial carcinoma cells (47). The STAM gene has also been characterized as an autophagy-related, signature gene (48), whereas elevated levels of TIMM23 (also known as Tim23) were observed in cell lines from patients with a common type of mitochondrial dysfunction, suggesting that upregulation of TIMM23 may be part of a compensatory response to this disorder, which is also responsive to rapamycin treatment (49). Owing to cross talk between mTORC1 and mTORC2 and our more limited knowledge of signaling downstream of mTORC2 (36), further studies potentially involving next-generation mTOR inhibitors designed to overcome cellular resistance mechanisms (50, 51) will be required to clearly associate components of the MLN128-induced translatome with mTOR in one or both of its complexes.

In conclusion, we have presented an experimental method designed to identify and quantify nascent proteins synthesized during a 2-h time frame in proliferating cells. Moreover, we showed that OPP labeling was able to capture rapid changes in the translatome upon acute perturbation that were otherwise undetectable in the whole cellular proteome. By incorporating the Dde biotin-azide cleavable linker into our OPP-ID protocol, we were able to enhance our understanding of the translational program impacted by acute MLN128 inhibition of mTOR by reducing unwanted background and improving our detection of low-abundance nascent proteins. These advantages of OPP-ID_CL_ will be most beneficial in situations where OPP labeling is expected to be low, such as when using short OPP labeling times or targeting specific cell types for labeling with caged OPP. Further improvements to the method are envisioned, such as direct capture of OPP-labeled samples on Dde agarose-azide or miniaturization of the protocol aided by automation. Indeed, a nascent proteomics workflow incorporating AHA labeling, a Dde biotin-DBCO cleavable linker for click chemistry, and high-throughput automation of the enrichment, wash, and elution steps was recently reported that elegantly demonstrates the feasibility of moving in this direction (52). Such improvements in sample handling may allow us to reveal currently inaccessible translatomes (*e.g.*, nascent proteins in single cells) by mass spectrometry going forward.

## Data Availability

The raw MS/MS files associated with this report have been deposited at the MassIVE site (https://massive.ucsd.edu) with the identifier MSV000089950. Annotated spectra can be viewed with the MS-Viewer tool in Protein Prospector (https://prospector.ucsf.edu/prospector/mshome.htm) with the Search Keys or URLs given in supplemental Table S8, where a list of the raw file names and sample descriptions is also provided.

## Supplemental data

This article contains supplemental Figures and Tables.
