# Supplementary material for: Capture, Release, and Identification of Newly Synthesized Proteins for Improved Profiling of Functional Translatomes
Source: Mol Cell Proteomics. 2023 Jan 13;22(3):100497. doi: 10.1016/j.mcpro.2023.100497 (PMC9971285; doi:10.1016/j.mcpro.2023.100497)
Supplement: Supplemental Figures [file mmc9.pdf]

Supplemental Figures for:

Capture, Release, and Identification of Newly Synthesized Proteins for Improved Profiling of Functional Translatomes

Nancy J. Phillips[1], Bala M. Vinaithirthan[1], Juan A. Oses-Prieto[1], Robert J. Chalkley[1], and Alma L. Burlingame[1,2]

1. Department of Pharmaceutical Chemistry, University of California, San Francisco, San Francisco, CA 94158, U.S.A.
2. Helen Diller Family Comprehensive Cancer Center, University of California, San Francisco, San Francisco, CA 94158, U.S.A

Corresponding Author: Alma L. Burlingame, Department of Pharmaceutical Chemistry, University of California San Francisco, Box 2240, 600 16th St, San Francisco, CA 94158. Tel: 415 476-5641. Email: alb@cgl.ucsf.edu

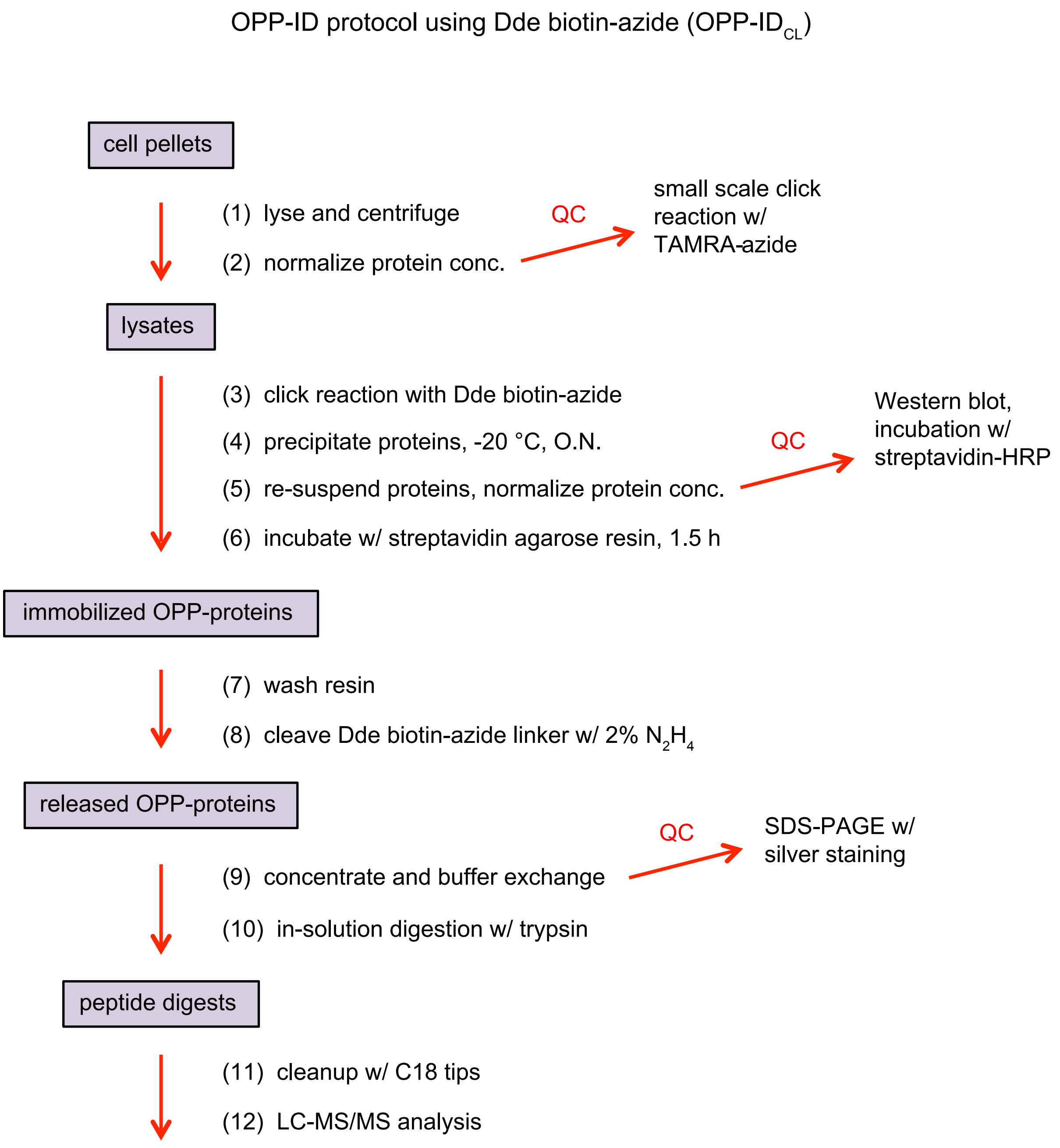

Supplemental Fig. S1. **Flow diagram of the OPP-ID protocol with incorporation of the Dde biotin-azide cleavable linker.** The major steps of the protocol are numbered on the diagram. Optional steps labeled as “QC” are quality control steps that can be performed to check the success of the protocol.

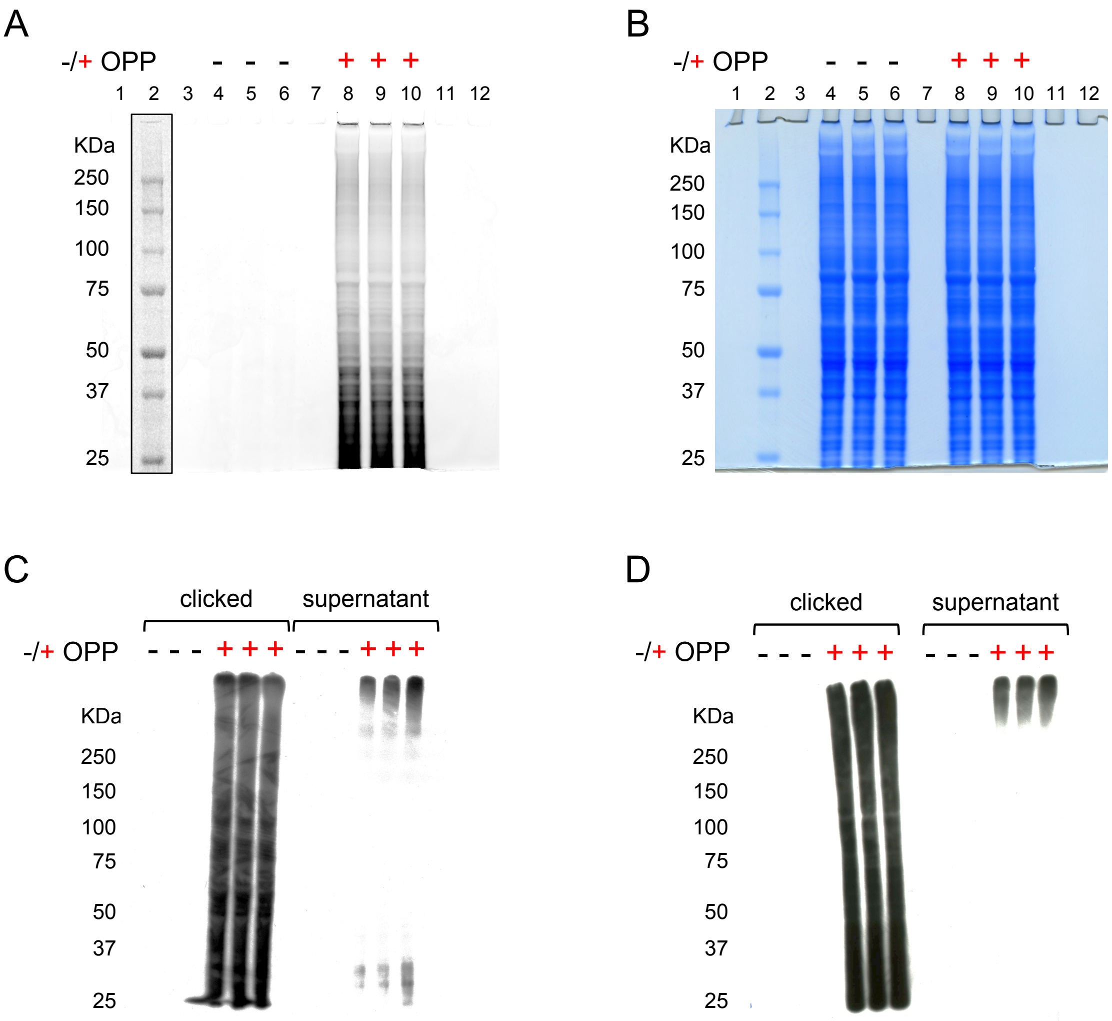

Supplemental Fig. S2. **SDS-PAGE gels of samples from the study comparing the OPP-ID and OPP-ID$_{CL}$ protocols.** (A) SDS-PAGE gel showing the six lysates used in this study after small-scale click reaction with TAMRA-azide, visualized with fluorescence detection. (B) The same gel as in (A) after Coomassie staining. (C) Western blot with streptavidin-HRP of clicked samples before and after capture on streptavidin magnetic beads in the OPP-ID protocol. (D) Western blot with streptavidin-HRP of clicked samples before and after capture on streptavidin agarose resin in the OPP-ID$_{CL}$ protocol.

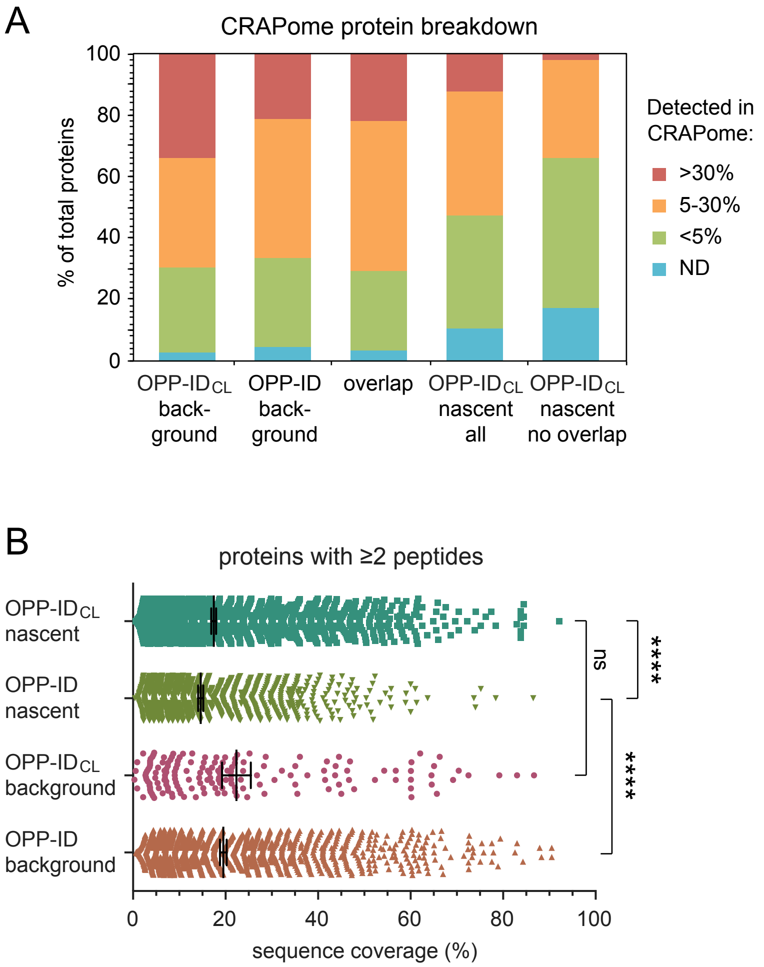

Supplemental Fig. S3. **Characterizations of the nascent and background proteins from the OPP-ID and OPP-ID$_{CL}$ protocols.** (A) Bar graph showing the proportions of identified proteins in the five indicated groups (OPP-ID$_{CL}$ background, N=243; OPP-ID background, N=2422; the overlap set found in both OPP-ID background and OPP-ID$_{CL}$ nascent, N=1911; OPP-ID$_{CL}$ all nascent, N=3749; and OPP-ID$_{CL}$ nascent minus the overlap set, N=1838) found in the CRAPome database at the specified frequencies. ND = not detected. (B) Scatter plot showing the protein sequence coverage observed in the background samples and the OPP-treated nascent protein pools (minus any overlapping background) from the merged replicates of the two protocols. The plot includes only proteins detected with ≥2 peptides in the condition (OPP-ID background, N=1752; OPP-ID$_{CL}$ background, N=167; OPP-ID nascent, N=1564; OPP-ID$_{CL}$ nascent, N=2802). Means and 95% confidence intervals are indicated on the plots (OPP-ID background, mean=19.5%; OPP-ID$_{CL}$ background, mean=22.4%; OPP-ID nascent, mean=14.6%; OPP-ID$_{CL}$ nascent, mean=17.4%). **** = p-value <0.0001, ns = not significant.

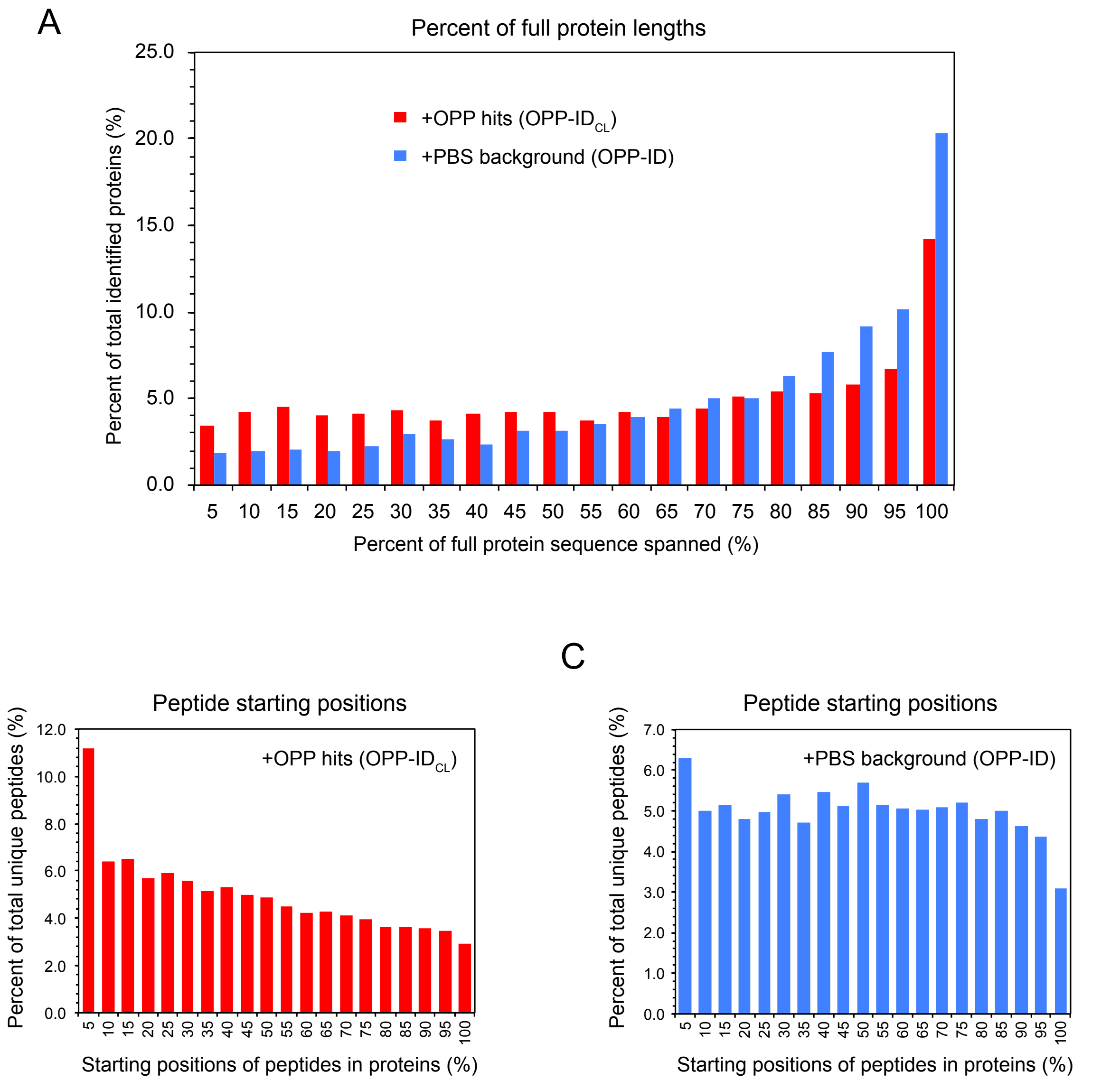

Supplemental Fig. S4. **Estimations of polypeptide lengths in background and nascent proteins.** These graphs compare background proteins from the OPP-ID experiment with PBS-treatment (N=2422) with nascent proteins (minus overlapping background) from the OPP-ID$_{CL}$ experiment with OPP-treatment (N=3749). (A) Unique peptides from each replicate were aligned to protein sequences to determine the minimal length of the protein sequence spanned in the corresponding polypeptides. The minimal polypeptide lengths were expressed as a percentage of the full protein lengths, the values binned as indicated, and normalized to equal 100%. (B and C) The starting positions (starting amino acid numbers) of all unique peptides from each replicate were expressed as a percentage of the full protein lengths, the values binned as indicated, and normalized to equal 100%. (B) Unique peptides from the nascent proteins from all replicates of the OPP-ID$_{CL}$ experiment. (C) Unique peptides from the background proteins from all replicates of the OPP-ID experiment.

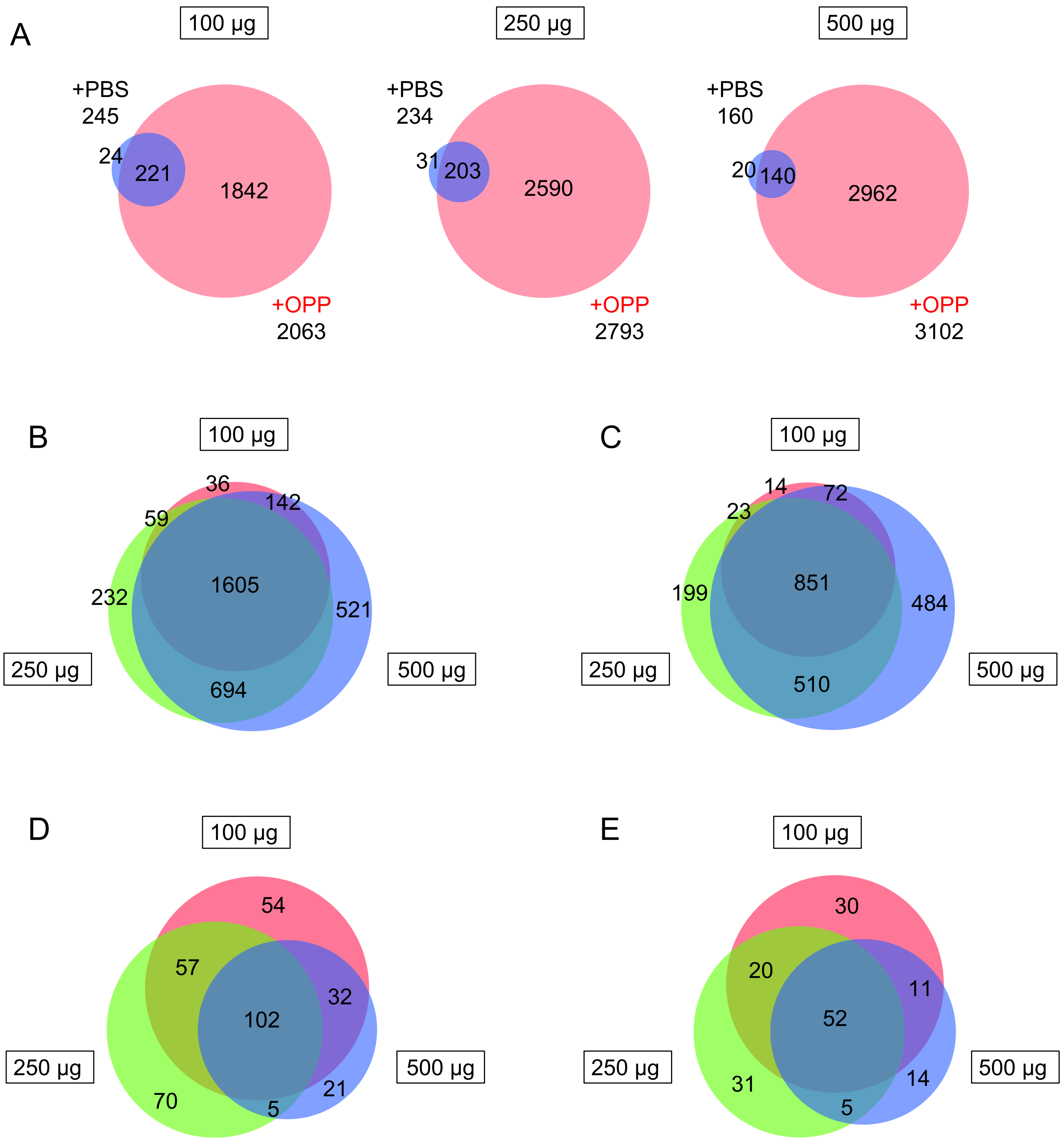

Supplemental Fig. S5. **Characterization of proteins detected at the three sample input levels.** (A) Venn diagrams showing the relative proportions of nascent and background proteins quantified at the three sample input levels. (B) Venn diagram showing the overlap of nascent proteins (minus any overlapping background) from the three sample input levels. (C) Venn diagram showing all proteins from (B) that were detected with ≥2 peptides in any biological replicate. (D) Venn diagram showing the overlap of background proteins from the three sample input levels. (E) Venn diagram showing all proteins from (D) that were detected with ≥2 peptides in any of the three biological replicates.

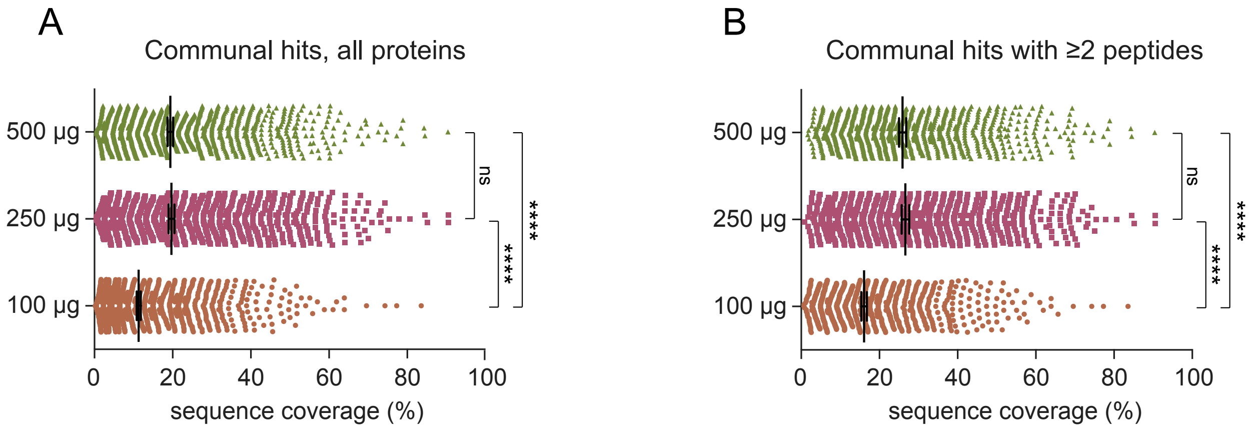

Supplemental Fig. S6. **Sequence coverage of proteins detected at the three sample input levels.** (A) Scatter plots showing the protein sequence coverage observed in the communal nascent proteins from the merged replicates of the three sample input sizes (N=1668). (B) Scatter plots as in (A), including only proteins detected with ≥2 peptides (N=1106). Means and 95% confidence intervals are indicated on the plots. **** = p-value <0.0001, ns = not significant.

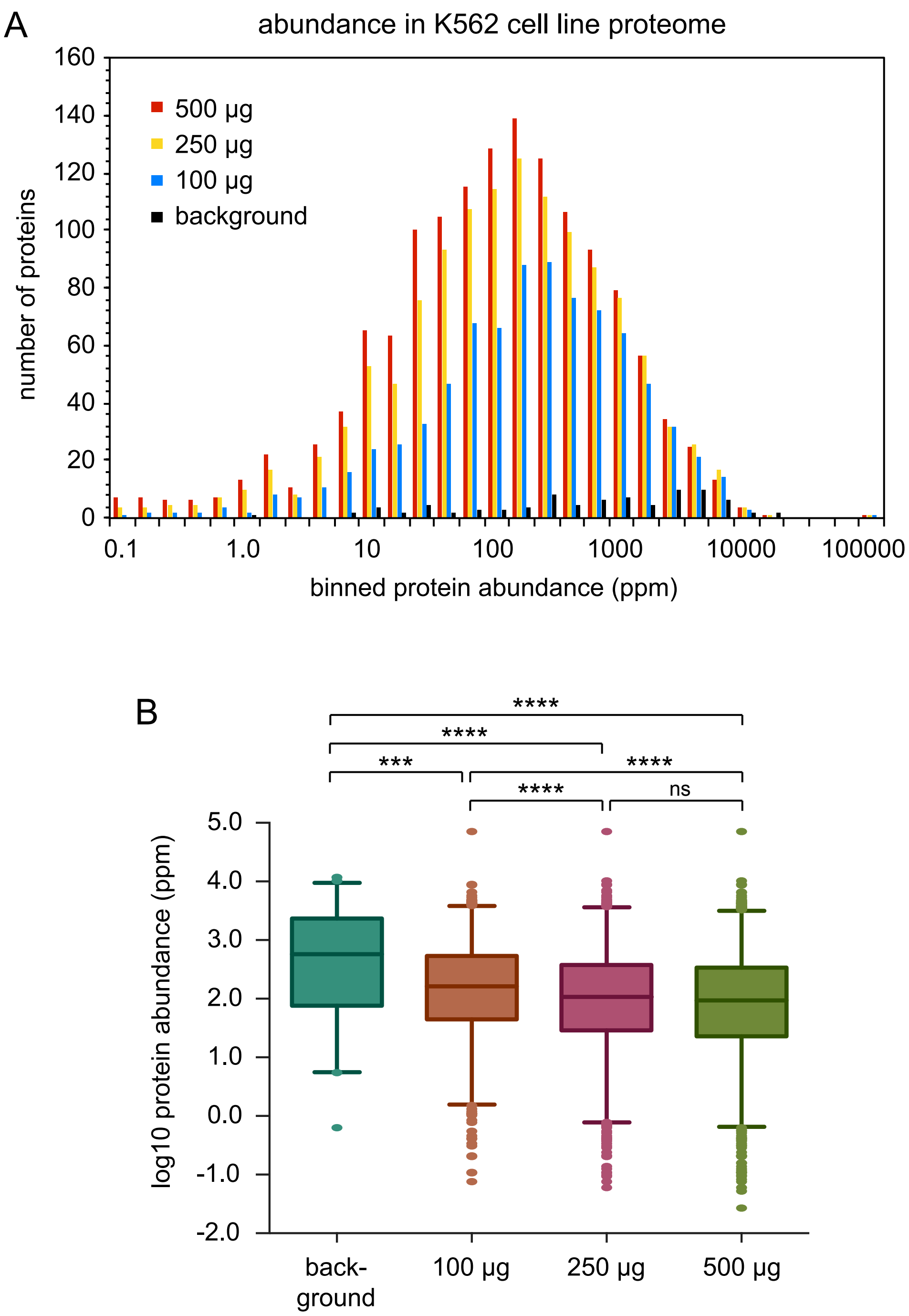

Supplemental Fig. S7. **Abundance distribution of our nascent proteins in the K562 proteome.** (A) Abundance values (ppm) for our nascent proteins at the three sample input levels were obtained from the PaxDb4.2 protein abundance database, binned, and plotted. Combined background proteins from all of the samples were also included. Proteins used in this analysis had ≥2 peptides in ≥1 biological replicate. (B) Log10 protein abundance values (ppm) for the samples as in (A), plotted as box and whiskers plots (background sample, N=87; 100 µg sample, N=827; 250 µg sample, N=1240; 500 µg sample, N=1394). The median protein abundance for each sample is indicated inside the box, and the whiskers represent the 2.5-97.5 percentile. **** = p-value <0.0001, *** = p-value <0.001, ns = not significant.

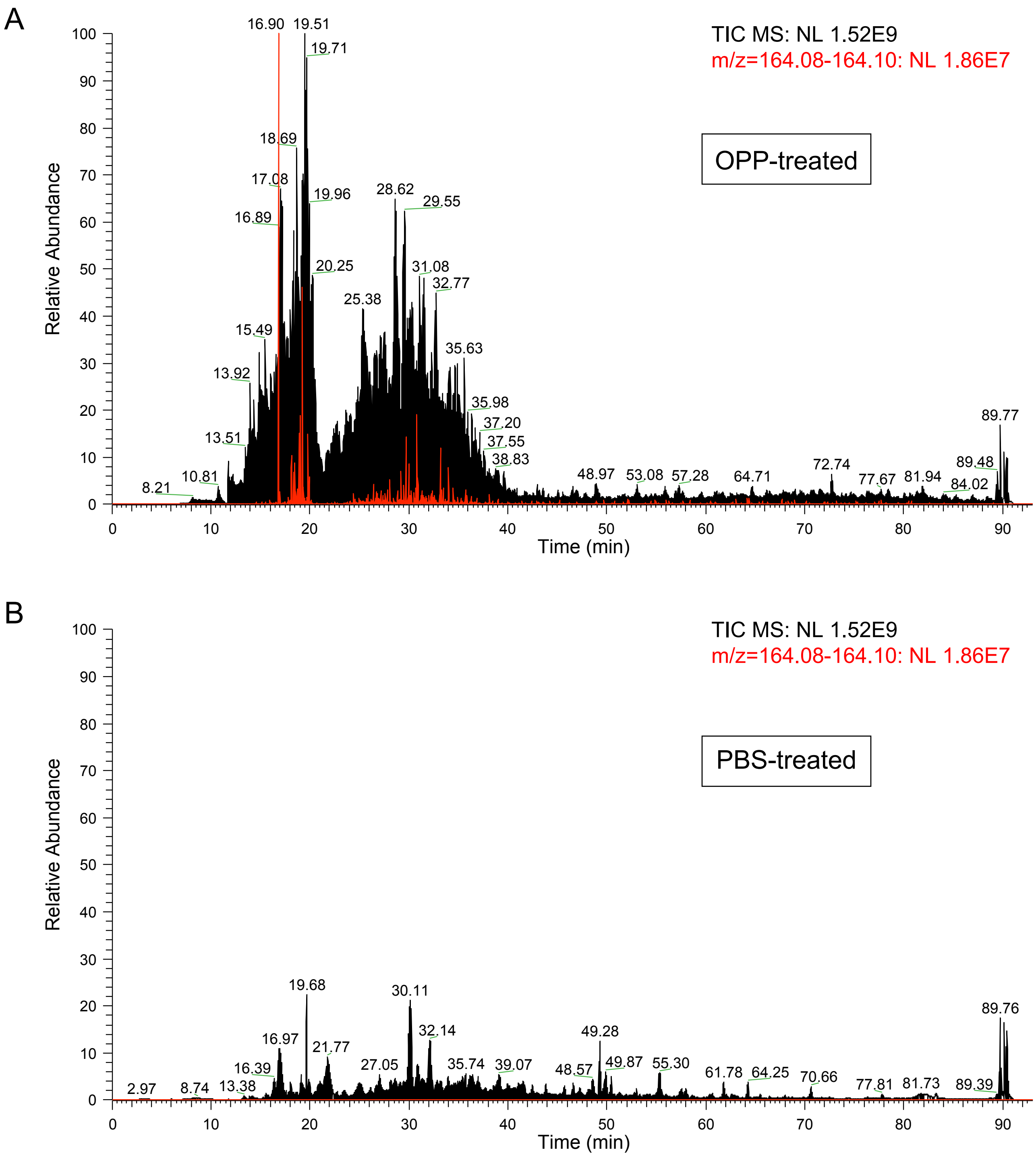

Supplemental Fig. S8. **Presence of the diagnostic purine fragment at m/z 164.09 arising from OPP-modified peptides.** (A) Overlapping TIC (black trace) and selected mass range m/z 164.08-164.10 (red trace), shown at the indicated normalized signal intensities, for the peptide digest from an OPP-treated sample. (B) Overlapping TIC (black trace) and selected mass range m/z 164.08-164.10 (red trace), shown at the same normalized signal intensities as in (A), for the peptide digest from a PBS-treated sample.

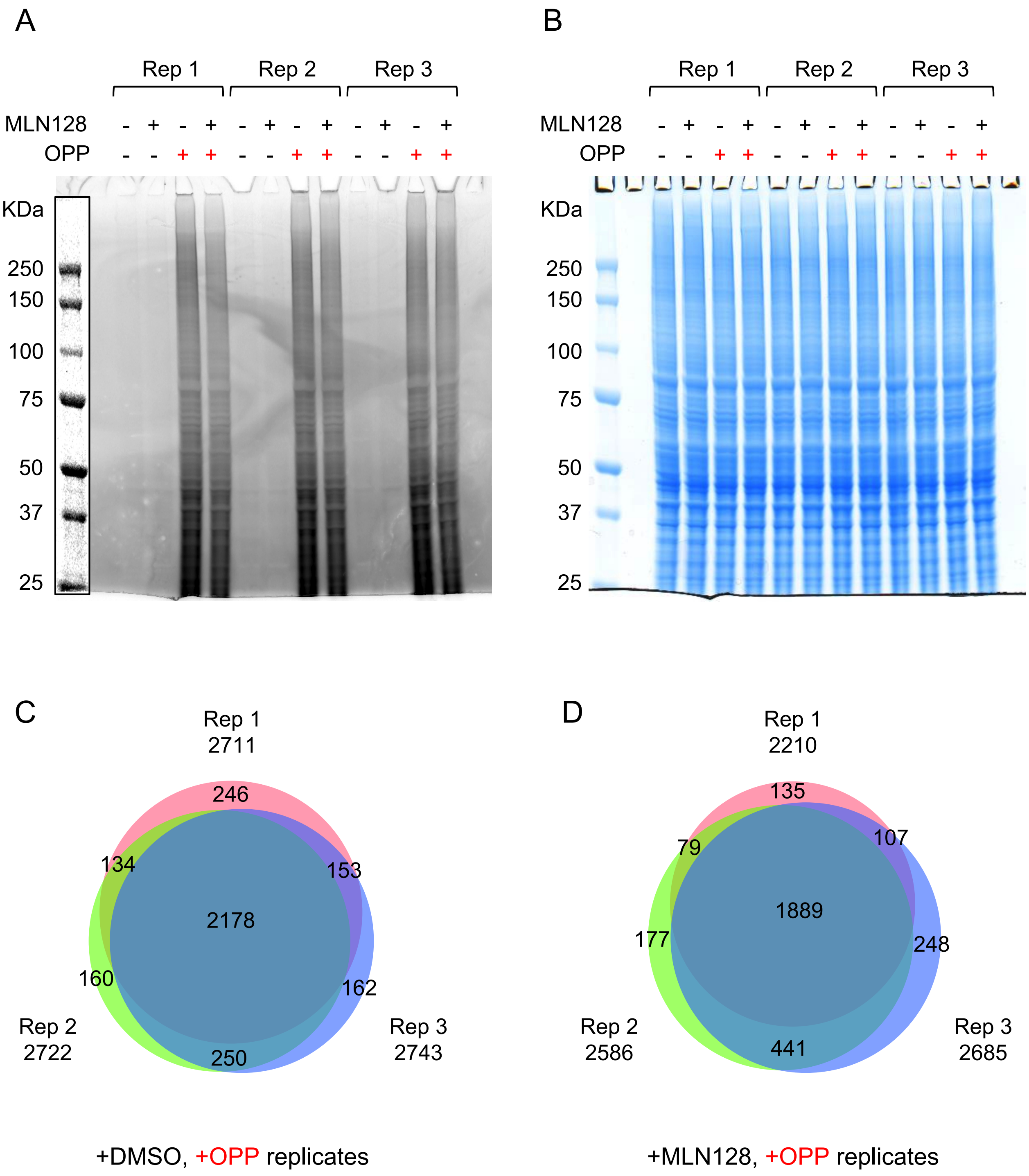

Supplemental Fig. S9. **K562 cells treated with MLN128, a potent inhibitor of mTOR.** (A) SDS-PAGE gel showing 12 lysates from this study after small-scale click reaction with TAMRA-azide, visualized with fluorescence detection. (B) The same gel as in (A) after Coomassie staining. (C) Venn diagram showing the overlap of proteins from the three biological replicates of the samples treated with DMSO and OPP. (D) Venn diagram showing the overlap of proteins from the three biological replicates of the samples treated with MLN128 and OPP.

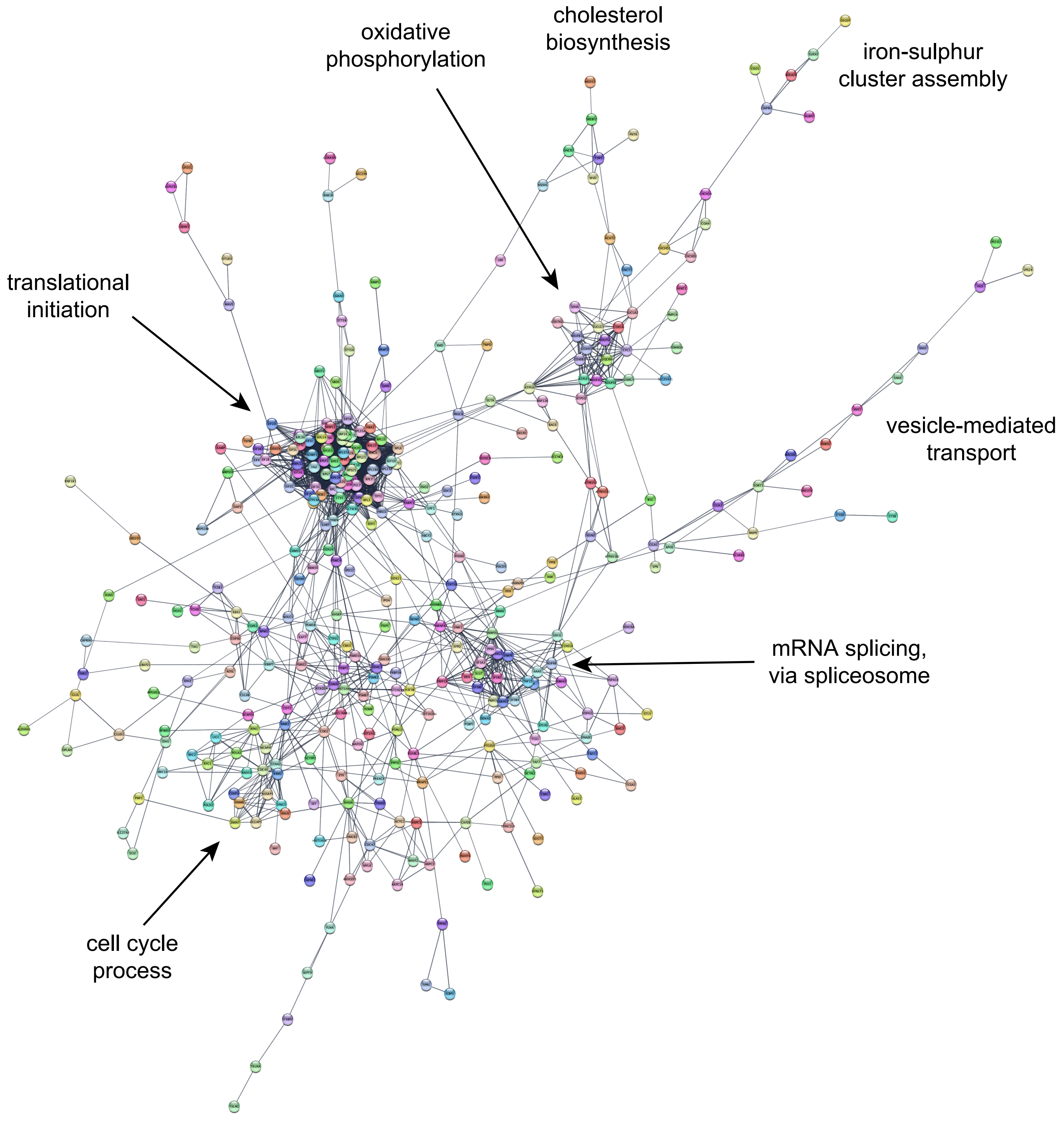

Supplemental Fig. S10. **Network of proteins downregulated with log2 FC <-1 upon treatment of K562 cells with MLN128.** The network of 351 functionally interacting proteins was generated from our full list of 519 downregulated proteins using the STRING app in Cytoscape (https://cytoscape.org/), with a high confidence (score) cutoff (set to 0.80). The GO biological processes associated with the indicated network clusters are given on the image.

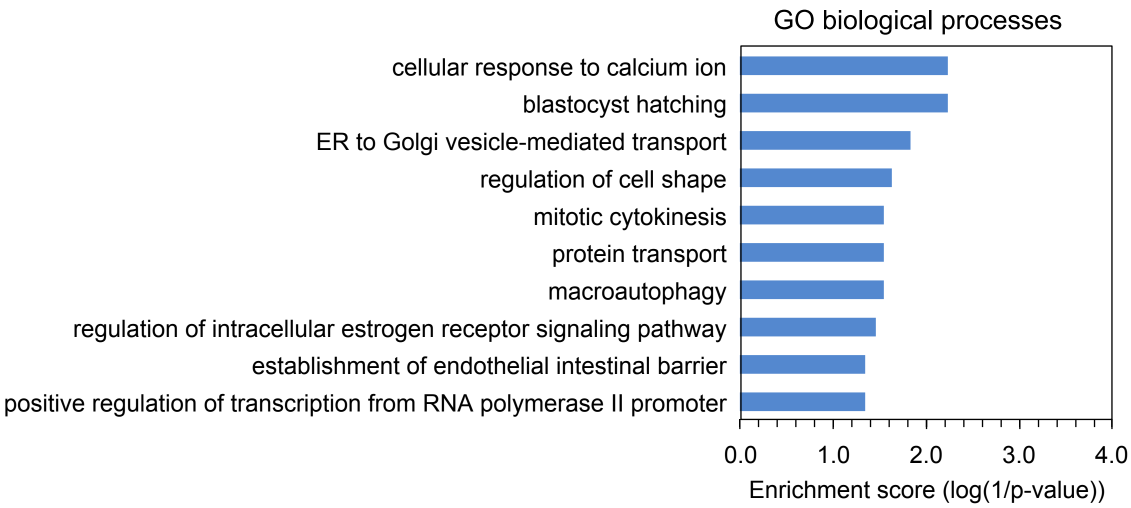

Supplemental Fig. S11. **Gene Ontology (GO) analysis of proteins upregulated upon treatment of K562 cells with MLN128.** GO biological processes enriched in the subset of 84 upregulated proteins with log2 FC >1 found in the nascent proteome upon inhibition of mTOR with MLN128.

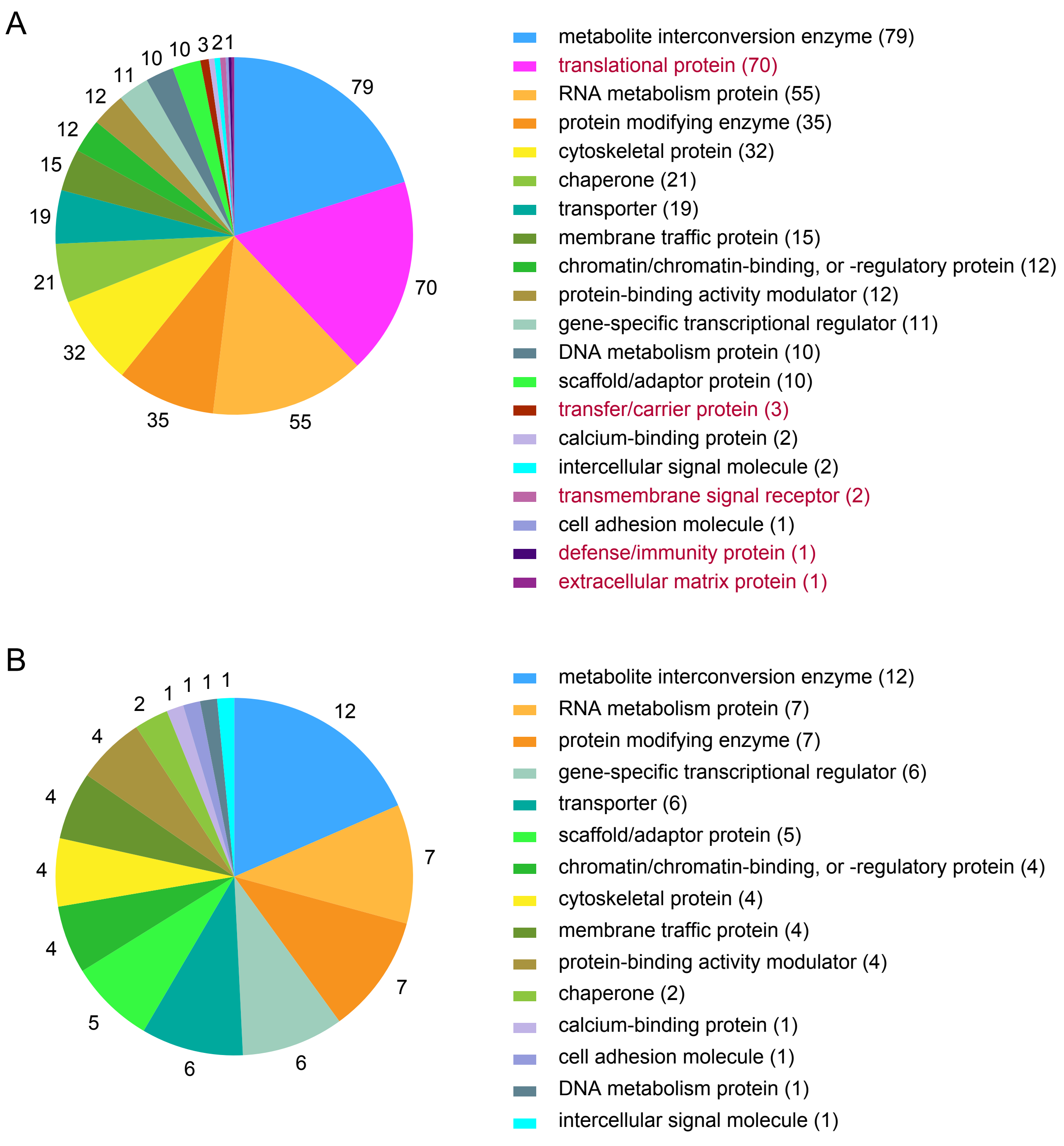

Supplemental Fig. S12. **PANTHER functional classifications of proteins differentially expressed with |log2 FC| >1 upon treatment of K562 cells with MLN128.** Protein accession numbers were submitted to PANTHER17.0 (http://www.pantherdb.org/) for classification. (A) Classifications of the downregulated proteins (393 of 519 proteins classified). (B) Classifications of the upregulated proteins (65 of 84 proteins classified). Functional categories given in red font were found only in the downregulated proteins.

A

Proteins ID'd	DMSO, OPP			MLN128, OPP		
	Rep 1	Rep 2	Rep 3	Rep 1	Rep 2	Rep 3
Per replicate	5047	5014	4883	4911	4930	4940
Combined	5464			5419		

C

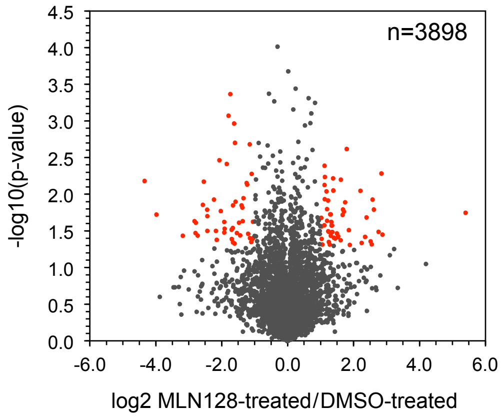

B

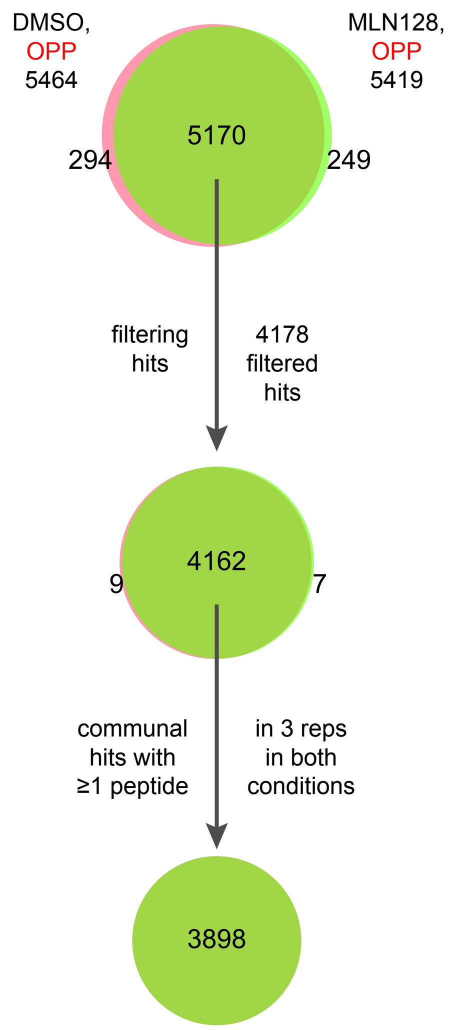

D

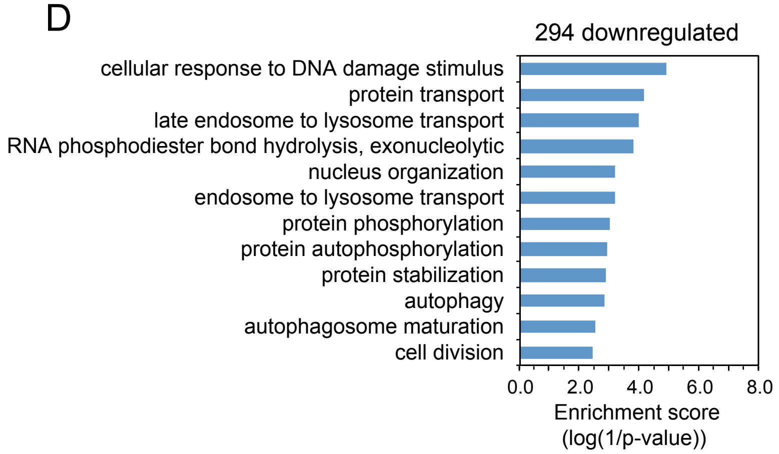

E

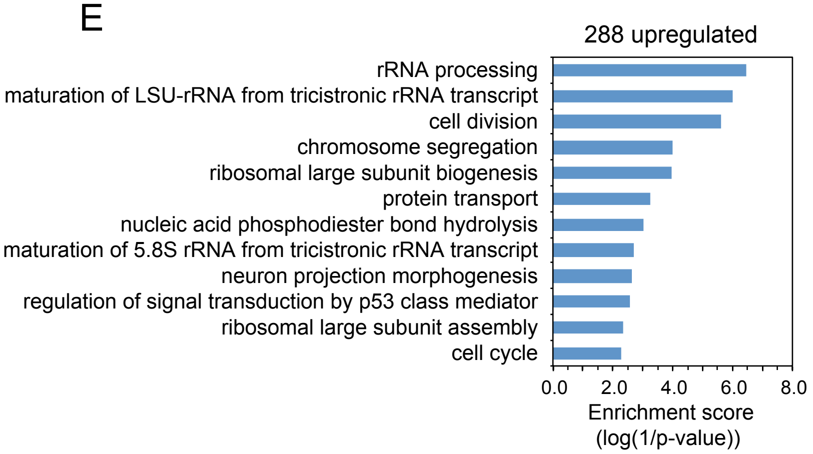

Supplemental Fig. S13. **Whole proteome analysis.** (A) Table summarizing the numbers of proteins quantified in all replicates of both OPP treatment conditions. (B) Venn diagrams showing the overlap of all proteins in the two samples conditions (+/- MLN128) and the process of filtering for hits with ≥2 peptides in ≥2 biological replicates in either condition. (C) Volcano plot of the 3898 communal hits, showing -log10(p-value) vs. protein fold change (log2) in MLN128-treated/DMSO-treated cells. Red dots represent statistically significant hits with |log2 FC| >1 and p-values <0.05. (D) GO biological processes enriched in proteins with log2 FC <-1. (E) GO biological processes enriched in proteins with log2 FC>1.
